# Weaponizing human EGF-containing fibulin-like extracellular matrix protein 1 (EFEMP1) for 21^st^ century cancer therapeutics

**DOI:** 10.18632/oncoscience.306

**Published:** 2016-05-23

**Authors:** Yi-Hong Zhou, Yuanjie Hu, Liping Yu, Chao Ke, Christopher Vo, Hao Hsu, Zhenzhi Li, Anne T. Di Donato, Abhishek Chaturbedi, Ji Won Hwang, Eric R. Siegel, Mark E. Linskey

**Affiliations:** ^1^ Neurosurgery, Brain Tumor Research Laboratory, University of California Irvine, Irvine, CA, USA; ^2^ Real-Time PCR, Ziren Research LLC, Irvine, CA, USA; ^3^ Neurosurgery, State Key Laboratory of Oncology in South China and Collaborative Innovation Center for Cancer Medicine, Sun Yat-sen University Cancer Center, Guangzhou, China; ^4^ Department of Biostatistics, University of Arkansas for Medical Sciences, Little Rock, AR, USA

**Keywords:** EFEMP1, EGFR, NOTCH1, MMP2, cell invasion, glioma, tumor cell subpopulation, orthotopic tumors, tumor vascularization

## Abstract

De-regulated *EFEMP1* gene expression in solid tumors has been widely reported with conflicting roles. We dissected EFEMP1 to identify domains responsible for its cell context-dependent dual functions, with the goal being to construct an EFEMP1-derived tumor-suppressor protein (ETSP) that lacked tumor-promoting function. Exon/intron boundaries of *EFEMP1* were used as boundaries of functional modules in constructing EFEMP1 variants, with removal of various module(s), and/or mutating an amino acid residue to convert a weak integrin binding-site into a strong one. A series of *in vitro* assays on cancerous features, and subcutaneous and intracranial xenograft-formation assays, were carried out for effects from overexpression of wild-type and variant forms of EFEMP1 in two glioma subpopulations characterized as tumor mass-forming cells (TMCs) or stem-like tumor initiating cells (STICs), where EFEMP1 showed cellcontext- dependent dual functions. One of the EFEMP1 variants was identified as the sought-after ETSP, which had a stronger tumor-suppression function in TMCs by targeting EGFR and angiogenesis, and a new tumor-suppression function in STICs by targeting NOTCH signaling and MMP2-mediated cell invasion. Therefore, ETSP may form the basis for further important research to develop a novel cancer therapy to treat many types of cancer by its tumor suppressor effect in the extracellular matrix compartment.

## INTRODUCTION

Fibulins are secreted glycoproteins that contain a series of epidermal growth factor (EGF)-like modules followed by a carboxy-terminal fibulin-type module, and function as intramolecular bridges within the extracellular matrix that mediate cellular processes and tissue remodeling. Neoplastic transformation could be looked upon as the crossroad of distortions in the extracellular microenvironment; hence, fibulins are commonly deregulated in cancer [1–3]. EFEMP1 (known as fibulin 3) is a fibulin of recent evolutionary origin in higher vertebrates. It was initially identified as a senescence protein [4, 5], and recently was widely reported in nearly all kinds of cancer to have deregulated expression, mainly via promoter hyper-methylations that correlated with its anti-cancer effect (see review in [6]).

The anti-tumor effects of EFEMP1 in most cancer types are most likely accounted for by its anti-angiogenic effect [7], its anti-EGFR/AKT-mediated growth signaling effect [8–11], and its anti-IGF1R [12] and/or anti-TGFbeta [13] mediated epithelial-to-mesenchymal transition effects, as well as its function in normal tissue to inhibit the expression and activities of matrix metalloproteinase [14]. EFEMP1's tumor-promoting role has been related to its pro-invasive role from activation of NOTCH [15] and/or NK-kappa B [16] signaling. Our recent studies have shown that both types of EFEMP1 roles operate in a cell-context-dependent manner in two tumor-cell subpopulations with differential activation of EGFR or NOTCH signaling, which stabilized the cell subpopulation equilibrium in responses to changes of the *in vivo* growth environment [17].

The reported involvements of EFEMP1 in cancer, and its site of action in the extracellular compartment [9], suggested the possibility of developing an EFEMP1- derived tumor-suppressor protein (ETSP) that would suppress both growth and invasion phenotypes of cancer and the extracellular cancer-supporting microenvironments. We report in this study the identification of such an ETSP from fourteen expression constructs of EFEMP1 having a deletion and/or mutation, based on effects generated from their over-expression, *in vitro* and *in vivo*, in two syngeneic glioma cell lines. These two glioma cell lines, U251 and U251-NS, respectively contain a preponderance of cells characterized with phenotype of tumor-mass-forming cells (TMC) or stem-like tumor initiating cells (STIC), and have the ability to inter-convert via mis-segregation of chromosome 7 to maintain tumor heterogeneity and optimal equilibrium of cell subpopulations benefiting overall tumor growth [18]. Overexpression of EFEMP1 nearly abolished intracranial tumorigenicity of TMC-enriched U251 [9], however, EFEMP1 overexpression in STIC-enriched U251-NS promoted cancer stem cell features [17]. ETSP has not only enhanced tumor suppression effect in U251, but also gained tumor suppression effect in U251-NS.

## RESULTS

### Identification of boundaries of functional modules for constructing EFEMP1 variants

**Figure 1:**
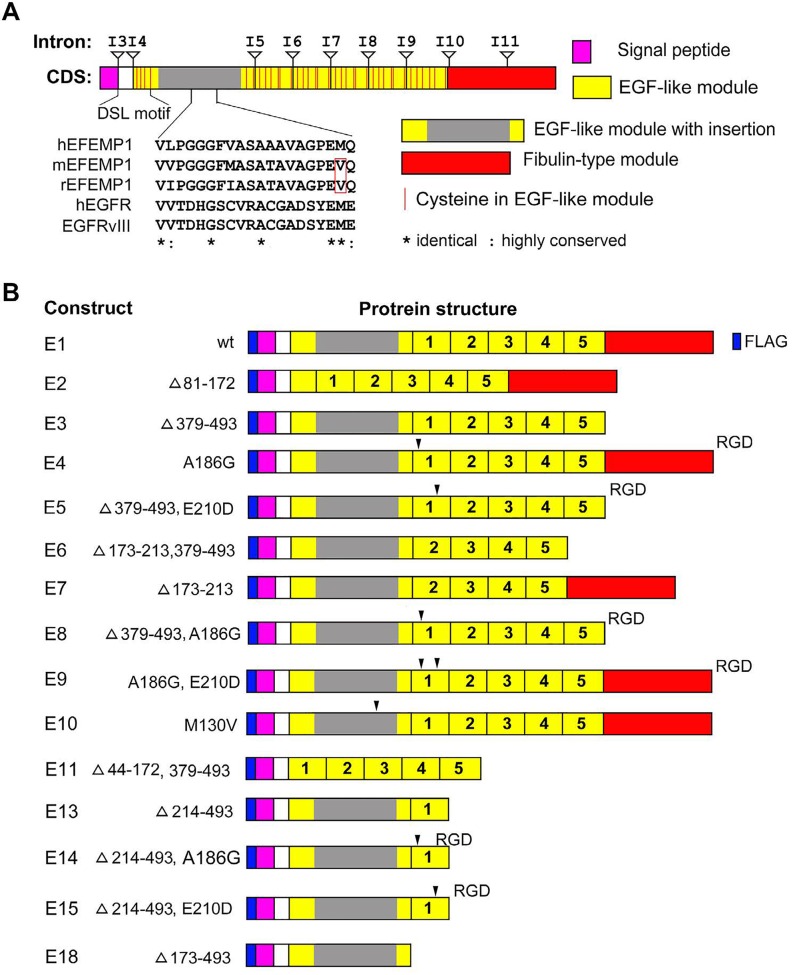
Depictures of wild-type and engineered variants of EFEMP1 **A.** alignment of *EFEMP1* mRNA coding sequence (CDS) with boundaries of exons and introns to functional modules of EFEMP1 protein. An EGFR-homologous region (EHR) uniquely occurs in EFEMP1 was shown, with multiple sequence alignment showing conserved residues between EHR of EFEMP1 and extracellular region of EGFR. **B.** schematicmodularillustration of wild-type (wt) and variant EFEMP1 proteins E1, E2, E3, E4, E5, E6, E7, E8, E9, E10, E11, E13, E14, E15, and E18. All were made to express protein carrying *N*-terminal FLAG-tag in order to examine their expressions.

A BLAST search with human EFEMP1 protein sequences showed that an ortholog was lacking in nonmammalian species. Further alignment of exon-intron boundaries of human EFEMP1 showed the precise locations of the boundary of EGF-like modules [6], suggesting intragenic exon duplications during evolution of the *EFEMP1* gene. Hence the five tandem repeats of EGF-like models in EFEMP1 may be positively selected in the evolution of mammals. Amino acidsequence alignment of three human fibulins (EFEMP1, EFEMP2, and FBLN5) showed an insertion following a short *N*-terminal region (presumably a signal peptide) that is unique to EFEMP1, so this module was denoted as “EGF-like module with insertion”. Further search for sequence homology with this EFEMP1-specific region in fibulin identified a region with twenty residues that has high sequence similarity between EFEMP1 and EGFR. The EGFR-homologous region (EHR) of EFEMP1 was aligned to the extracellular region of EGFR of all four isoforms of EGFR and an EGFR deletion mutant EGFRvIII (Figure 1A).

According to boundaries of exons and introns of the *EFEMP1* gene, PCR primers were designed to amplify cDNA fragments of EFEMP1, and to construct EFEMP1 variants with removal of one or more modules in constructs of E2, E3, E4, E5, E6, E7, E8, E9, E10, E11, E13, E14, E15, and E18, yielding modular structures of protein as depicted in Figure 1B. Precise protein and nucleotide sequences of all engineered variants and wild-type (wt) of EFEMP1, including the FLAG tag at the *N*-terminal and 5′-end, respectively, are presented as supplemental data (Tables S1 and S2).

Multiple protein sequence alignments of human EFEMP1, EFEMP2, and FBLN5 also identified in FBLN5 a strong integrin-binding site RGD in the first EGF-like module following the signal peptide, which does not occur in EFEMP1 and EFEMP2. However, two weak integrinbinding sites (RAD and RGE) are in the EFEMP1 EGFlike module with insertion, at locations of 186 and 210, respectively. Hence, base-pair mutations were designed in the PCR primers to convert RAD, RGE, or (in one case) both into RGD for constructing the EFEMP1 variants of E4, E5, E8, E9, E14, and E15, in addition to deletions of one or more modules. One amino acid difference between human and rodent was identified in the 20-aa EHR of EFEMP1 (Figure 1A), hence a mutation of M130V was made to convert EHR in EFEMP1 of human to rodent for constructing the EFEMP1 variant E10 (Figure 1B).

### Identification EFEMP1-derived tumor suppressive protein (ETSP)

FLAG-tagged, wild-type EFEMP1 protein construct (E1) and FLAG-tagged EFEMP1 protein-variant constructs were made in mammalian expression vector pcDNA3.1+ and lentiviral vector pTRIPZ, then transfected or infected, respectively, into glioma cell lines U251 and its clonal neural sphere culture U251-NS. Functional assay then were performed to determine the effects from overexpression of these different variants of EFEMP1, in comparison to empty vector and wt EFEMP1. U251 was chosen to screen EFEMP1 variants for ETSP that retains the parental protein's tumor suppressor function in high proliferating TMC via targeting EGFR and angiogenesis, as demonstrated in our previous studies [9, 11]. U251-NS was chosen to screen EFEMP1 variants for ETSP without the parental protein's pro-invasive function in STIC [17]. These two cell subpopulations, which were enriched in U251 and U251-NS, different in genetic and phenotypic features and able to cooperate in tumor formation [18]. Hence the ETSP identified based on these two syngeneic glioma cells shall have broad tumor suppression effects that could also be applied in other cancer models.

Using an absolute real-time qRT-PCR system [19], the level of transgene overexpression in the transfected/infected cells was determined, as shown in Table 1. To detect the specific transcripts from expression constructs, the forward primer was designed to anneal to the FLAG tag and the reverse primer to anneal to exon 4 of *EFEMP1*. The results of Table 1 indicate the following: (A) that the E1, E2, E3, E4, E5, E6, E7, E8, E9, E10, E11, E13, E14, and E15 constructs were expressed in plasmidtransfected U251 cells; (B) that the E1, E2, E5, E6, E7, E8, E11, E13, E15, and E18 constructs are expressed in lentivirus-infected U251 cells, in a doxycycline-inducible manner; and (C) that the E1, E2, E3, E5, E7, E8, E10, E11, E13, E15, and E18 constructs are expressed in lentivirusinfected U251-NS cells, with addition of doxycycline.

**Table 1:** qRT-PCR detection of transgene overexpression

	U251 pcDNA	U251 pTRPZ		U251-NS pTRPZ
		(−) Dox	(+) Dox	(+) Dox
Vector	N/E	0	0	0.1
E1	1.6	0.1	12.7	133.3
E2	23.7	0.1	3.4	74.2
E3	81	N/E	N/E	46.7
E4	1.6	N/E	N/E	N/E
E5	22.4	0.1	4.5	1.9
E6	29.6	0.1	8.1	N/E
E7	1.8	0	3.3	43.8
E8	1.6	0.1	5.2	14.2
E9	1.4	N/E	N/E	N/E
E10	49.5	N/E	N/E	55.2
E11	153.9	0.1	54.9	198
E13	24.2	0.1	25.4	6.6
E14	10.6	N/E	N/E	46.7
E15	62.5	0.1	9.3	85.4
E18	N/E	0.2	6.9	142.3

Values are ratio of FLAG-*EFEMP1/ACTB* times 1000

N/E: Not examined

### *In vitro* functional assays to narrow the list of ETSP candidates

**Figure 2:**
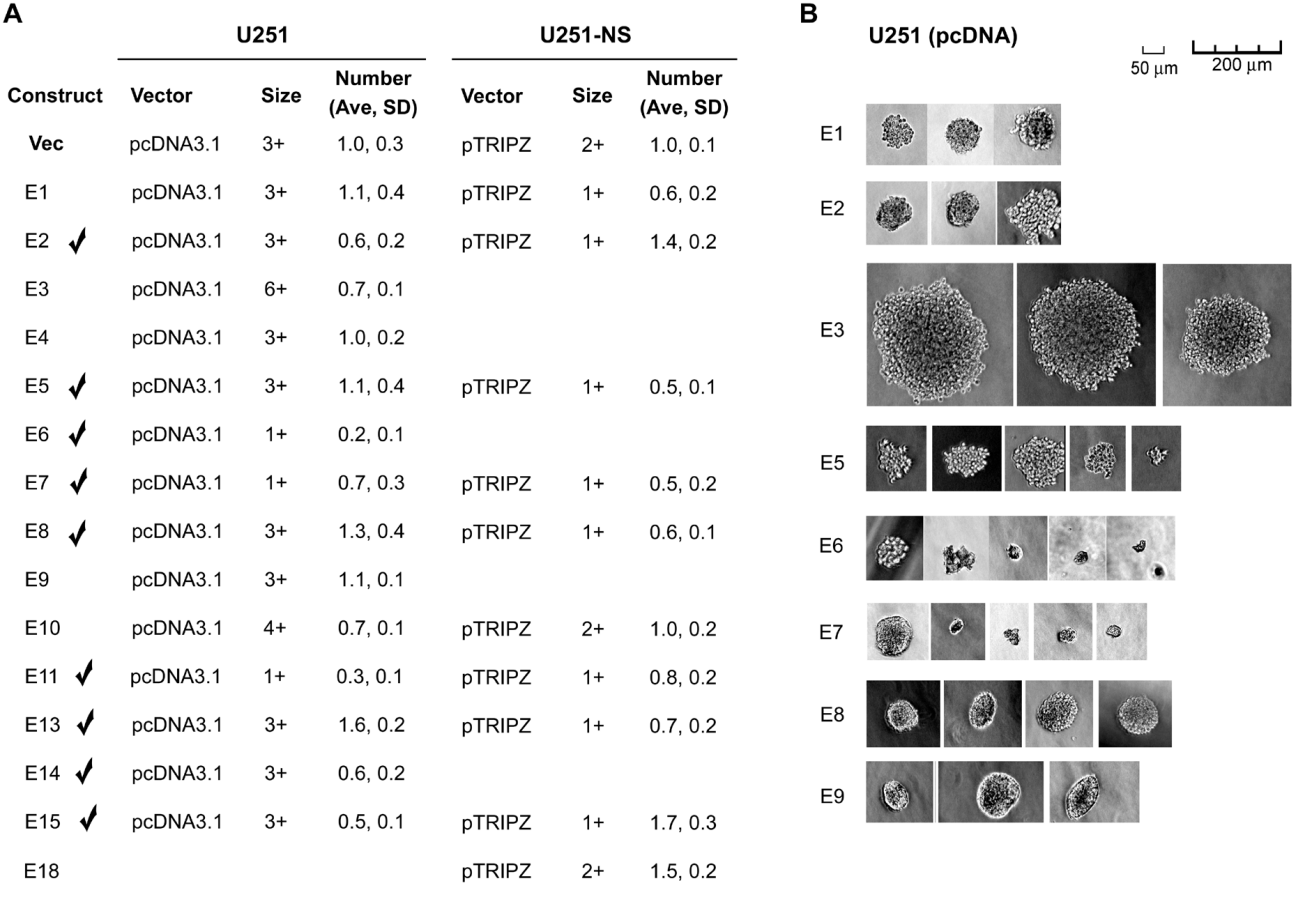
Soft agar colony formation assay **A.** colony number of U251 stable transfectants and U251-NS lentiviral infectants of wild-type EFEMP1 and EFEMP1 variants, normalized to the average number of colonies from the Vec control (set at unity). The average (Ave) and standard deviation (SD) were based on colonies formed in 4 individual wells. Representative colonies (5-10), were measured and scaled by the diameter of the colony: 1+ (~10 μm), 2+ (~20 μm), 3+ (30-40 μm), 4+ (40-60 μm), 5+ (50-100 μm), 6+ (100-200 μm). **B.** representative images of colonies from U251 stable transfectants.

The soft agar colony-formation assay is a commonly used test to determine cancerous phenotypes. For monolayer culture of U251, it will determine the effect of EFEMP1 and its variants on anchorage-independent growth ability, while for neurosphere culture of U251-NS, it will determine the effect on neurosphere formation. The effect of EFEMP1 and its variants in U251 on anchorageindependent growth was examined with U251 stably transfected with the E1, E2, E3, E4, E5, E6, E7, E8, E9, E10, E11, E13, E14, and E15 pcDNA 3.1+ constructs, and compared to U251 stably transfected with plasmid vector pcDNA 3.1+ (Vec). As shown in Figure 2A (left panel) and Figure 2B, EFEMP1 variants expressed from constructs E2, E6, E7, E11, E14, and E15 showed their effects on reducing colony formation rate without increasing colony size in soft agar by U251. In contrast, EFEMP1 variant E3 markedly increased U251 colony size in soft agar, with an average 10-fold increase in diameters of the colonies, compared to that of Vec. EFEMP1 variant E10 slightly increased soft agar colony size while decreasing colony number, while variant E13 increased colony number 60% without increasing colony size. The effect of EFEMP1 and its variants in U251-NS on neurosphere formation was examined with U251-NS infected with the E1, E2, E5, E7, E8, E10, E11, E13, E15, and E18 pTRIPZ lentiviral constructs and compared to U251-NS infected with pTRIPZ lentiviral vector (Vec). As shown in Figure 2A (right panel), EFEMP1 variants E1, E5, E7, E8, E11, E13, and E15 showed their effects on reducing colony formation rate and/or colony size in soft agar by U251-NS. Hence, based on results of soft agar colony formation in both U251 and U251-NS, nine candidates of ETSP (E2, E5, E6, E7, E8, E11, E13, E14, and E15) were selected as marked in Figure 2A for other functional analyses.

**Figure 3:**
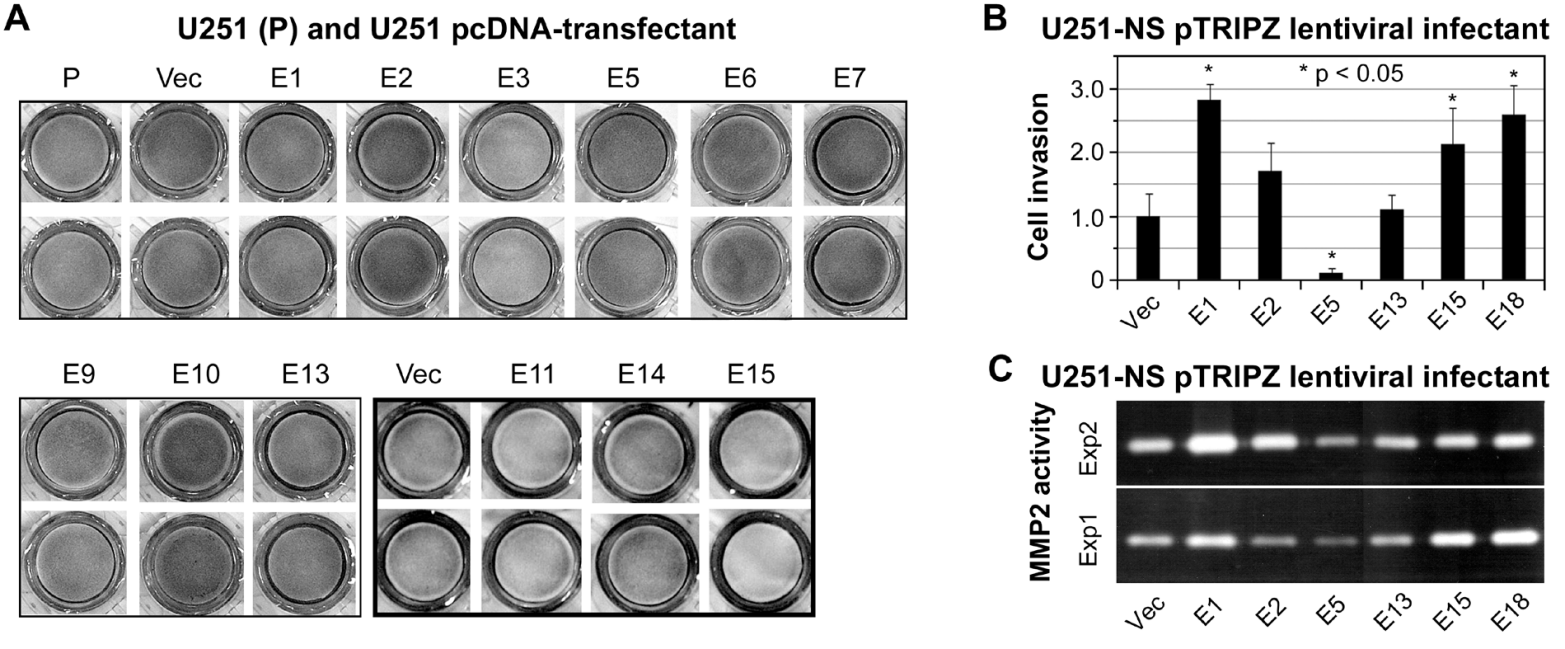
Detection of cell invasiveness **A.** a photograph of bottom layers in transwells from a matrigel cell invasion assay, with duplicates of cells of U251 stable transfectants of wild-type EFEMP1 and EFEMP1 variants. Invasion pictures from two independent experiments were shown by think and thick-line boxes. **B.** a plot of normalized cell counts in bottom layers of transwells from a matrigel cell invasion assay, with triplicates of cells of U251-NS lentiviral infectants of wild-type EFEMP1 and EFEMP1 variants, following a 3-day culture in doxycycline-containing medium. **C.** a photograph of two gels of the zymography assay, conducted with cells from the matrigel invasion assay described in panel B.

The matrigel cell invasion assay is frequently used to detect cancer cell invasiveness and the gelatin zymography assay is used to detect matrix metallopeptidase secreted by cancer cells and activated in extracellular compartment. Our prior study showed MMP2-depedent invasion of U251 [20], and EFEMP1's pro-invasive effect on U251-NS based on results of matrigel cell invasion and gelatin zymography assays [17]. Hence ETSP candidates were tested for their ability to inhibit cell invasiveness of both U251 and U251-NS. Our initial matrigel assays were shown in Figure 3A on U251 stable-transfectants. It showed that constructs E3, E11, and E15 markedly suppressed the invasiveness of U251 cells. In contrast, EFEMP1 variants expressed from constructs E2, E7, and E10 increased the invasiveness of U251 cells. Matrigel invasion assays was then carried out for U251-NS infected with the E1, E2, E5, E13, E15, and E18 pTRIPZ lentiviral constructs, following a 3-day induction of transgene expression by doxycycline. As shown in Figure 3B, EFEMP1 protein variant expressed from construct E5 significantly inhibited U251-NS cell invasion; whereas the wild-type EFEMP1 and EFEMP1 variants expressed from constructs E1, E15, and E18 significantly promoted U251-NS cell invasion. Matrigel invasion assays was also carried out for U251 infected with the E1, E2, E5, E6, E7, E8, E10, E13, E15, and E18 pTRIPZ lentiviral constructs, following a 3-day induction of transgene expression by doxycycline. Unlike U251-NS, no significant difference on invasiveness of U251 following a transient overexpression of wild-type EFEMP1 and all the examined EFEMP1 variants (data not shown). The differential effects on cell invasion of stabletransfected U251 shown in Figure 3A are likely due to long-term overexpression of different EFEMP1 variants and/or clonal effects of stable transfectants.

The gelatin zymography assay was then carried out using protein precipitated from conditioned medium of U251-NS infectants, which were used for invasion assay with results showing in Figure 3C. MMP2 is a protease highly expressed by glioma cells and facilitates cell invasion. Our prior study showed higher expression of MMP2 and active level of extracellular MMP2 by U251-NS, in comparison of U251 [18]. The results of zymography assays showed that the EFEMP1 protein variant expressed from construct E5 significantly inhibited U251-NS glioma cells from making activated MMP2, whereas the wild-type EFEMP1 and EFEMP1 variants expressed from constructs E1, E15, and E18 significantly promoted this activity. In U251, there lacks consistent results from repeated experiments showing an effect on MMP2 by wild-type EFEMP1 or any of the examined EFEMP1 variants (data not shown), which are consistent with their lacking effects on matrigel invasiveness. Our prior studies demonstrated the highly invasive nature of U251-NS in comparison to U251, and EFEMP1 promoted U251-NS invasion [17, 18]. Hence, EFEMP1 variants that gained the function of suppressing invasiveness of U251- NS met the criteria of ETSP to target invasive STIC.

### Subcutaneous xenograft formation assay to identify ETSP with angiostatic function

**Figure 4:**
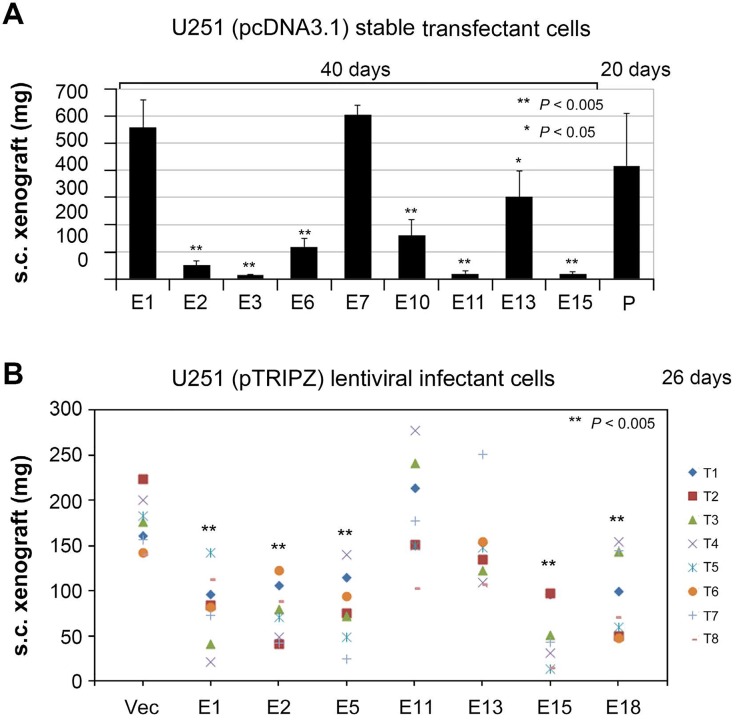
Subcutaneous (s.c.) xenograft formation assay of U251 **A.** comparison of s.c. tumor weights of U251 stable transfectants of wild-type EFEMP1 and EFEMP1 variants. P, parental un-transfected U251. **B.** comparison of s.c. tumor weights of U251 lentiviral infectants of pTRIPZ-lentiviral constructs of vector, wild-type and variants of EFEMP1.

We have demonstrated that U251-NS cells lacked subcutaneous (s.c.) tumorigenicity, but gained s.c. tumorigenicity with overexpression of pro-angiogenic protein VEGF-165 [18]. Hence, the s.c. xenograft formation assay has the power to specifically detect the ability of cancer cells on inducing angiogenesis. To identify constructs of ETSP that maintain the function of the parental protein EFEMP1 in suppressing angiogenesis in the s.c. xenograft model [9], the s.c. xenograft formation assay was performed with U251 (P) and U251 stable transfectants having E1, E2, E3, E6, E7, E10, E11, E13, and E15 pcDNA 3.1+ constructs. The results verified the effect of wild-type EFEMP1 in suppressing s.c. tumorigenicity of U251 as seen in our prior study [9], namely that it took nearly double the amount of time for U251 transfected with the E1 construct to develop s.c. xenografts of similar weight compared to the un-transfected parental cell. As shown in Figure 4A, all the examined EFEMP1 variants (E2, E3, E6, E10, E11, E13, and E15) except E7 showed an enhanced effect in U251 on suppressing s.c. tumor growth.

We then carried out the s.c. xenograft assay with U251 infectants of E1, E2, E5, E11, E13, E15, and E18 pTRIPZ lentiviral constructs and empty construct of pTRIPZ (Vec). Mice were fed with doxycycline-containing water from day 1 and throughout the experiment. RFP expression was shown in the resulting tumors (data not shown), suggesting the success of Dox induction of transgene expression in formation of s.c. xenografts. As shown in Figure 4B, compared to the vector control, wildtype EFEMP1 and EFEMP1 variants expressed from constructs E2, E5, E15, and E18 all suppress formation of U251 s.c. xenografts.

### Intracranial xenograft formation assay

**Figure 5:**
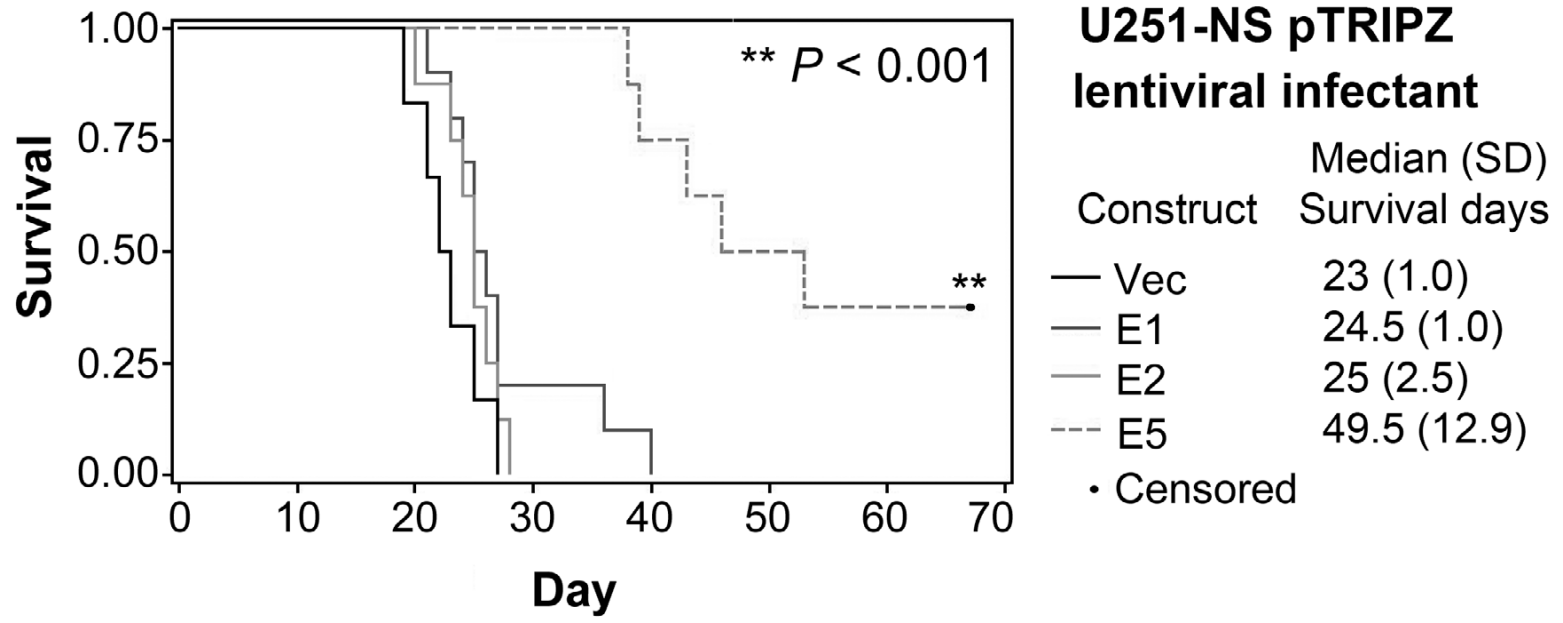
Intracranial (i.c.) xenograft formation assay of U251-NS Kaplan-Meier survival curves were was plotted from overall survivals of mice following i.c. implantation of U251-NS infectants of pTRIPZ-lentiviral constructs of vector, wild-type and variants of EFEMP1.

Combining results described above from *in vitro* growth and invasion assays and s.c. xenograft assay aiming to identify an ETSP that functions to suppress tumor initiation, tumor growth, and tumor invasion, it came down to EFEMP1 variant E5, which was further examined using an orthotropic tumor model. We then carried out intracranial (i.c.) implantation of U251-NS pTRIPZ-infectants of E1, E2, and E5 and empty vector in nude mice. Mice were fed with doxycycline-containing water from day 1 and throughout the experiment. RFP expression was shown in the resulting tumors, suggesting the success of Dox induction of transgene expression in formation of i.c. xenografts. As shown in Figure 5, the median survival time of mice from i.c. implantation of U251-NS infectant of EFEMP1 variant E5 was about doubled compared to that from implantation of U251- NS infectants of wild-type EFEMP1 and empty vector. Although the variant EFEMP1 protein expressed from the E2 construct inhibited s.c. tumorigenicity, it had no significant effect on i.c. tumorigenicity as compared to infectants of wild-type EFEMP1 and empty vector.

### Mechanisms of action of ETSP by targeting EGFR and NOTCH

**Figure 6:**
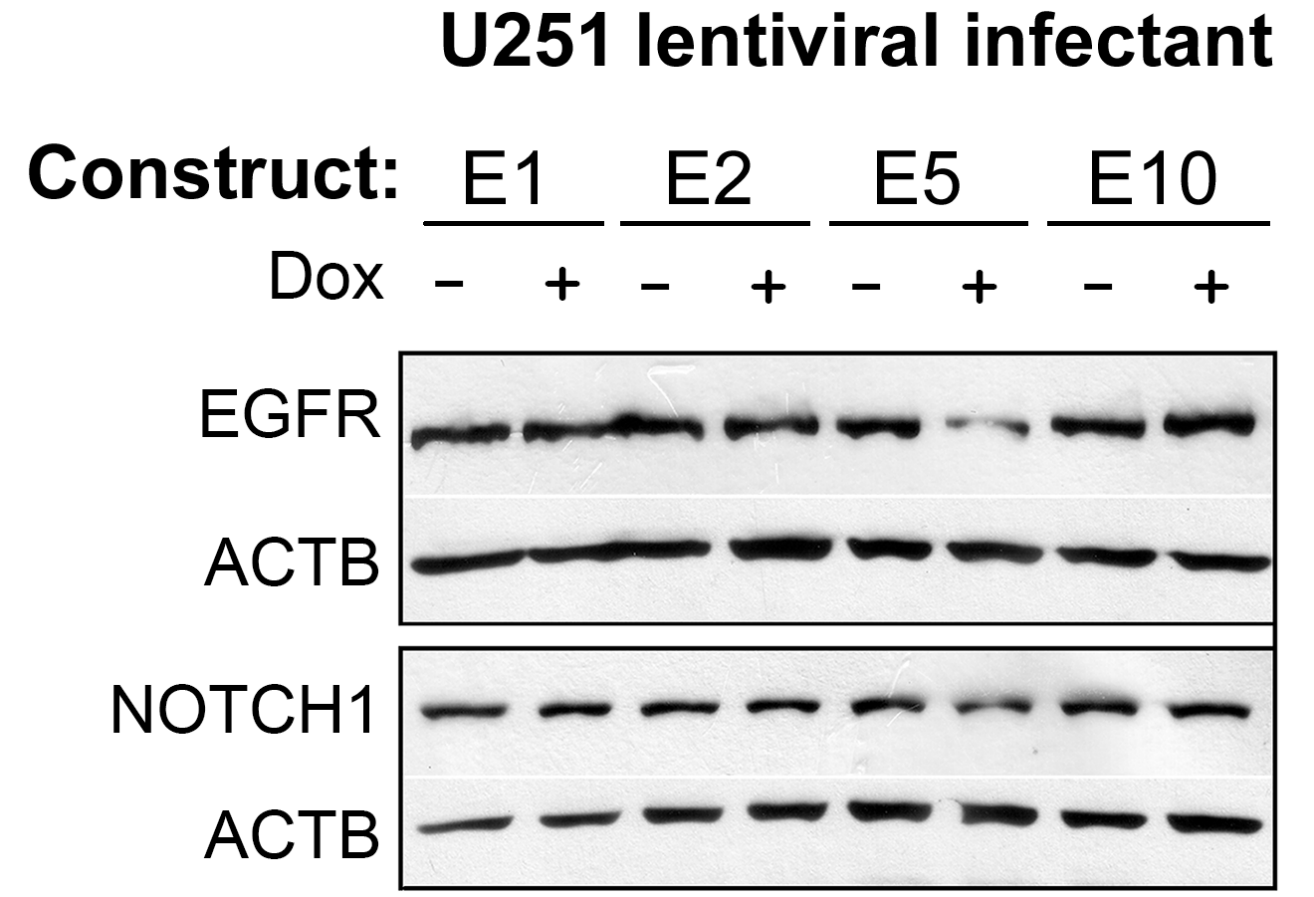
Immunoblotting EGFR and NOTCH 1 Whole cell lysate of U251infectants of pTRIPZ-lentiviral constructs of wild-type and variants of EFEMP1 following a 3-day cultures in medium with or without addition of doxycycline (Dox) were blotted and detected by antibodies of EGFR and NOTCH1. ACTB was detected to control equal protein loading. Since the transduced cells were kept in the “off” condition by omitting doxycycline, the un-induced cells served as the negative control for induced cells. In addition, construct E10, the 1-bp altered variant of EFEMP1 that showed minimal functional alteration of EFEMP1, was included as a negative control.

Nearly half of gliomas overexpress EGFR, which has been functionally related to enhanced tumor cell survival and growth. EFEMP1 was shown to reduce membrane EGFR level in U251, but this effect was barely detectable in immunoblots of whole cell lysate [11], as shown in immunoblotting conducted with parental culture U251 cells infected with the E1 variant (Figure 6). In this immunoblotting assay, whole-cell lysate of pTRIPZlentiviral infectants (E1, E2, E5, and E10) were extracted from cells cultured in medium containing doxycycline to induce transgene expression for 3 days, and the co-expressed RFP was shown 1 day after under a fluorescent microscope, suggesting expression of transgene for at least of 2 days. As shown in Figure 6, transient overexpression of EFEMP1 variant E5, but not the other two variants or the wild-type version of EFEMP1, reduced the total level of EGFR.

Activation of NOTCH signaling is a common feature of cancer stem cells. Both U251-NS and U251 cells express NOTCH1. Here in the same whole cell lysate of U251 lentiviral infectants used for blotting EGFR protein, NOTCH1 protein was examined and showed reduction due to a transient expression of EFEMP1 variant E5. Hence the identified ETSP from expression of EFEMP1 variant construct E5 would give stronger tumor suppression than the parental EFEMP1 in TMC by targeting EGFR and angiogenesis, and also would gain a new tumor-suppression function by targeting NOTCH. Furthermore, ETSP from expression of EFEMP1 variant construct E5 would have gained tumor-suppression function in SITC by targeting NOTCH signaling and MMP2-related oncogenic activities and the corresponding function in tumor invasion and cancer stem cell phenotypes. Taken together, the data and results set forth above indicate that the EFEMP1 protein variant E5 is a potent ETSP.

## DISCUSSION

The deadly form of brain cancer, glioblastoma multiforme (GBM), for which there is not yet any effective treatment, is made up of disparate subpopulations of cells characterized by having distinct proliferation and infiltration properties and high antigenic properties. In the past 40 years, only a modest 2.5-month increase in median survivals has been achieved for patients with GBM, by adding concomitant temozolomide treatment to radiotherapy after surgery. Although most GBMs are found to have activation of EGFR with its regulated growth-signaling activities, there is no reported success in clinical trials with therapeutants that target EGFR, neither as a monotherapy nor in combination with other agents [21]. There is an urgent need to develop innovative and effective anti-brain-cancer therapeutants, having the ability to target the malignant nature of cancer cells as well as the tumor's microenvironment. This is the objective of the present study, which has been achieved.

Conflicting roles of EFEMP1 in cancer have been reported, including dual effects in two different tumor cell subpopulations [17]. EFEMP1's tumor-suppression property is indicated by the following. EFEMP1 has an anti-angiogenic function via suppression of endothelialcell sprouting [7]. Reduced EFEMP1 expression and/or EFEMP1 promoter methylation occurs in lung, liver, breast, colon, prostate, and nasopharyngeal carcinoma [8, 22–27]. EFEMP1 expression in GBM, hepatocellular and nasopharyngeal carcinoma is correlated with a favorable prognosis [8, 9, 23, 24]. EFEMP1 suppresses EGFR/AKT signaling [8, 9, 11] and TGF-β or IGF1R signaling [12, 13]. EFEMP1's contrasting oncogenic function is indicated by the following. Elevated EFEMP1 expression has been correlated to poor prognosis for cervical cancer [28] and ovarian carcinoma [29]. In pancreatic adenocarcinoma cells, EFEMP1 activates AKT signaling in pancreatic carcinoma cell lines [30], and activates NF-κB signaling to promotes the migration and invasion of osteosarcoma via MMP-2 with induction by AEG-1 [16]. In certain glioma cells with activation of NTOCH signaling, EFEMP1 has been shown to enhance *in vitro* substrate-specific cell adhesion and promote cell motility and dispersion [31].

Based on functional assays on tumor suppression activities, a protein modified from wild-type EFEMP1 was obtained, which we have named ESTP (EFEMP1- derived tumor suppressor protein). Given that all the wild-type EFEMP1 and EFEMP1 variants were made to carry an *N*-terminal signal peptide, we assume that the protein products of wild-type EFEMP1 and EFEMP1 variants made by the host cells were secreted into the extracellular compartment to affect all the examined cell phenotypes. It is rational to assume that a protein product made using the sequence of an ESTP lacking a signal peptide would exhibit its therapeutic effect in the tumor's extracellular environment following direct administration into a tumor. The therapeutic effect of a protein product based on the sequence of the ESTP has been proven in a glioma animal model, as well as mechanistically, by its targeting of EGFR, NOTCH, MMP2 and pAKT) (manuscript in preparation, abstract ATPS-99 and poster shown at the 20^th^ SNO Annual Scientific Meeting and Education Day, San Antonio, Nov. 19-22, 2015). Further research on the therapeutic application of ESTP should translate into a novel cancer therapy characterized by modulating, in cancer cells, the activity and/or expression level of proteins that include EGFR, AKT, NOTCH, and likely protein tyrosine kinase 2 (PTK2), due to obtaining a strong integrin binding-site. Based on the mechanisms of action of the ETSP, the cancer can be a glioma of low, medium and high grade, a fibrosarcoma, a colorectal cancer, a lung cancer, a colon cancer, a liver cancer, a breast cancer, a prostate cancer, or a nasopharyngeal cancer. And the cancer cell can be a cancer stem-like cell or a tumor mass-forming cancer cell.

## MATERIALS AND METHODS

### Animal use for subcutaneous (s.c.) and intracranial (i.c.) xenografts and analysis

The animal experiments were approved by the Animal Care and Use Committee (IACUC) of University of California, Irvine. For studies using subcutaneous (s.c.) xenografts, cells (1 × 10^6^ cells / 50 μl DMEM/F12) were subcutaneously injected into nude mice, anterior to their right and left thighs, on both sides. Tumor measurements were taken every 3-4 days after implantation, and tumor volume was calculated using the formula V = (L*W^2^)/2 (L, length; W, width). Mice were euthanized at a predetermined time of the experiment or when tumor volume exceeded 1.5 cm^3^. For studies using intracranial (i.c.) xenografts, glioma cells (1 × 10^5^ / 3 μl DMEM/F12) were injected 3 mm into the frontal lobe, 2.5 – 3 mm right of the Lambda point and 1.8-2 mm anterior to the coronal suture, of 4 - 6-week-old female nude mice (stain NCrNu-M, Taconic, Hudson, NY) using a stereotactic frame with micro manipulator, and with injection speed (1.5 μl/min) controlled by a Harvard Apparatus Model 11 Plus syringe pump. Mice were observed daily, weighed 1-3 times a week, until moribund signs (hunchback posture and/or 20% weight loss) appeared, and were terminated the following day (which was recorded as the survival date).

Mice implanted with cells transduced by lentiviral vector of pTRIPZ (Vec, wild-type EFEMP1 or EFEMP1 variants), were given water containing 1 mg/mL Dox throughout the experiment. Overall survival of mice bearing intracranial glioma xenografts was estimated using Kaplan-Meier survival curves, and the *P* values were from Log-Rank statistics on pair-wise comparisons of the two groups. The significance level was set at *P*<0.01 in order to adjust for the multiple comparisons without overinflating Type II error. SAS versions 9.2 and 9.3 (The SAS Institute, Cary, NC) were used for all analyses.

### Glioma cell lines and culture conditions

The human glioma cell line U251 was obtained from Dr. Alfred Yung, M.D. Anderson Cancer Center, University of Texas. U251-NS was a clonal line of neural sphere culture of U251, as described in our prior study [18]. Cell line authentication for U251 and U251-NS were based on the genetic profiles (7-STR markers) by IDEXX RADIL, Columbia, MO), as shown in our previous study [18]. U251 with or without transfection with plasmid DNA or infection with lentivirus were cultured in DMEM/F12 medium supplemented with 5% bovine serum, U251- NS with or without transfection with plasmid DNA or infection with lentivirus were cultured in DMEM/F12 medium supplemented with EGF (20 ng/ml), bFGF (20 ng/ml), and 1% B27 (Invitrogen, Carlsbad, CA), in 37°C in humidified CO2 (5%) incubators.

### EFEMP1 deletion/mutation constructs, stable transfectants, and lentiviral infectants

All the expression constructs of full-length EFEMP1 having wild-type (wt) amino acid sequence and the deletion/mutation variants of EFEMP1 were constructed to carry a FLAG tag at the *N*-terminus by the FLAG-containing 5′ PCR primer. Expression constructs encoding the E1, E2, E3, E4, E5, E6, E7, E8, E9, E10, E11, E13, E14, E15, and E18 wild-type and variant EFEMP1 proteins illustrated in Figure 1B were made by PCR, with primers designed to create restriction sites for cloning and/or ligation of two separate cDNA fragments of *EFEMP1*. PCR products were cloned into TA-cloning vector pCR4.0 and sequence verified, prior to subcloning into mammalian expression vector pcDNA3.1+ (Life Technologies) and lentiviral vector pTRIPZ. A shuttle vector had been constructed so as to introduce an internal ribosome entry site for expression of EFEMP1 wildtype and variant proteins in pTRIPZ (Open Biosystems, Huntsville, AL) under the same promoter used for red fluorescent protein (RFP).

U251 stable transfectants were created by transfecting cells with plasmid DNA constructs in pcDNA3.1 and culturing them under selecting antibiotic (400 μg/ml neomycin), until surviving cells formed colonies. Individual colonies were picked using pipet tips, and transferred to 6-well plates to expand the colonies. U251 (parental and neural sphere cultures) lentiviral infectants were established by generating lentivirus from co-transfecting HEK293 with plasmid DNA constructs in pTRIPZ together with its derived constructs, psAX2, and pCMV-VSV-G. The lentiviral infectants were established after elimination of uninfected cells by a 1-2 week culture under selecting antibiotic (1.25 μg/ml puromycin), and addition of doxycycline (1μg/ml) to monitor success of infection via RFP expression in live cells with an inverted fluorescent microscope.

### Real-time absolute quantitative reverse transcription (AqRT-) PCR

RNA samples were extracted from glioma cell cultures using MiniPrep kits from Zymo Research (Irvine, CA). The cDNA samples were converted using Superscript III reverse transcriptase (Invitrogen) following manufacture's protocol, diluted more than 20 times with 10 mM Tris.HCl (pH7.5), then used (4 μl/each reaction) in real-time PCR, with SYBR Green I master mix from Roche (Indianapolis, IN). Four 10- fold serial dilutions of standard AG1097 (Ziren Research, Irvine, CA) containing target genes (including FLAG-tagged 5′-EFEMP1 cDNA fragment) and referent genes (*ACTB* and *GAPDH*) was included in each real-time PCR, with selection of the “Standard” approach and a defined standard quantity (10^5^, 10^4^, 10^3^, 10^2^ per reaction). Copy number of a gene's mRNA in the converted cDNA samples was normalized to copy number of *ACTB*, which were determined by the same standard, hence giving rise to an absolute ratio of two gene expressions as detailed in method of AqRTPCR [19]. AqRT-PCR primers for FLAG-containing EFEMP1 are 5′-ATGGACTACAAAGACGATGACG-3′ and 5′-TGCATTGCTGTCTCACAGGATC-3′ to amplified 151-bp PCR product, for ACTB are 5′-TCCTTCCTGGGCATGGAGT-3′ and 5′-TGATCTTCATTGTGCTGGGT-3′ to amplified 190-bp PCR product.

### Soft agar colony formation assay

Cells (800-1000) were mixed with 1 ml of 0.3% soft agar in DMEM/F12 medium, supplemented with 5% bovine serum or a mitogen supplement described above in “Glioma cell lines and culture conditions”, spread onto hardened 0.5% soft agar in the same medium (1 ml per well in four corner wells of a 6-well plate). 1 ml of the same medium was added 2 and 3 weeks later and colony numbers were counted 4 weeks later under a microscope with a 4X lens. The cells were U251 control cells and U251 cells stably transfected with constructs E1, E2, E3, E4, E5, E6, E7, E8, E9, E10, E11, E13, E14, E15, or pcDNA-empty vector (Vec). The cells were cultured in medium containing doxycycline to induce transgene expression. The total number of colonies in each well was counted under a microscope with a 4X lens.

### Matrigel invasion assay

Matrigel (9-12 μg/ml) was thawed on ice or at 4°C, 10-14 volumes of ice-cold serum-free medium were added to the thawed matrigel, 200 μl were added to transwell plates (12 wells; 8μm), and then incubated at room temp for >1 hour. Before plating cells, unbound material was gently aspirated off. The matrigel coating is colorless and not visible. Cells were prepared by detaching cells grown in 100 mm dishes to 80-90% confluency, by washing with 10 ml PBS, digesting with 2 ml 0.05% trypsin-EDTA for 30 sec, removing the trypsin, detaching the cells by tapping the bottle, then adding 10 ml culture medium and pipetting up and down, and finally transferring the cells to a 15-ml tube. The cells were then spun down, resuspended in 10-15 ml SF-medium, taking 10 μl to count cells with a hemocytometer. 5×10^6^ cells were then spun down and resuspended in 5 ml SF-medium at 1×10^6^/ml. 5 × 10^5^ cells were added to coated wells of the transwell plates, and 1 ml 0.05% calf serum-medium was added to the bottom well for U251 cells, and serum-free medium for U251-NS cells. Plating was done in a CO_2_ incubator, where plates were then cultured for 24 hours.

Cells that penetrated the matrigel were stained with HEMA3 CMS following the manufacturer's protocol. A Q-tip dipped in water was used to remove the matrigel membrane, which was then air-dried. The dry membrane was placed on a slide and Aqua mounting medium was added. A coverslip was then placed on top, and 10 pictures were taken for each filter with a microscope using a 20X lens. Cells were counted based on the pictures taken, compared to the control (Vec), which was set to unity, and the results analyzed by t-test (paired, two tailed).

### Gelatin zymography assay

Conditioned medium of lentiviral infectants of U251 or U251-NS, grown for 48 hours in serum-free DMFM/F12 medium containing 1 μg/ml doxycycline, was collected as follows. Cells were spun down, and 0.3 ml of supernatant were transferred into a 1.5 ml tube. Protein was precipitated from the supernatant by adding 1.2 ml cold acetone. The precipitate was spun immediately at 14K RPM for 5 min at 4°C. Conditioned-medium protein (CM-P) was resuspended in 100 μl 1x RIPA + 1x protease inhibitor cocktail. 2 μl of CM-P were taken to determine protein concentrate with a BIO-RAD Protein Assay. The resuspended CM-P was stored at 80°C. 2-4 μg of CM-P were mixed with, 1xRIPA, and 2x sample buffer (100 μl 10% BB, 10 ml glycerol, 1 g SDS, 7.5 ml 1 M Tris.HCl pH 6.8, with added sterile ddH2O to 50 ml), briefly spun, and loaded onto a zymography gel in 1x Tris-glycine-SDS running buffer. The gel was run at room temp @ 90 V for 2 hours. The gel was washed several times in 2.5% Triton X-100 for 1-2 hour at RT with gentle shaking. The gel was then incubated overnight at 37°C in protease reaction buffer (50 mM Tris (pH=7.5), 10 mM CaCl_2_, 150 mM NaCl). The gel was then stained for 30 min with Coomassie Blue R-250 (0.1% coomassie blue, 10% acetic acid, 10% isopropanol in ddH2O) with gentle shaking. The gel was then the stained in 10% acetic acid, 30% methanol solution three times for 20 minutes, and air-dried overnight between two cellophane papers.

### Immunoblotting

30-40 μg protein of whole cell lysate were loaded onto an 8% SDS-PAGE gel in 1x Tris-glycine-SDS running buffer. The gel was run at room temperature, 90 V, for 2 hours, and transferred to nitrocellulose membranes. The blots were blocked with 5% skim milk for 1 hour, then incubated in 1% BSA containing rabbit primary antibodies for EGFR (1:1000 dilution) or NOTCH1 (1:1000 dilution) overnight. Then they were washed with 1X TBST for 1 hour, incubated with anti-rabbit IgG HRP-conjugated secondary antibody (1:10,000 dilution) for 1 hour. An ECL detection kit was used to develop the signal in the immunoblots.

## SUPPLEMENTARY TABLES





## Abbreviation

ETSP: EFEMP1-derive tumor suppressor protein
TMC: tumor mass-forming cell
STIC: stem-like tumor initiating cell
HER: EGFR-homologous region in EFEMP1
s.c.: subcutaneous
i.c.: intracranial
