# Supplementary material for: Weaponizing human EGF-containing fibulin-like extracellular matrix protein 1 (EFEMP1) for 21st century cancer therapeutics
Source: Oncoscience. 2016 May 23;3(7-8):208–19. doi: 10.18632/oncoscience.306 (PMC5043071; doi:10.18632/oncoscience.306)
Supplement: Supplementary file 1 [file oncoscience-03-208-s001.pdf]

SUPPLEMENTARY AND TABLES

Supplementary Table S1: Multiple alignment of FLAG-tagged EFEMP1 wildtype (E1) and variant (E2 – E18) protein sequences

See Supplementary File

Supplementary Table S2: Multiple alignment of FLAG-tagged EFEMP1 wildtype and variant nucleotide sequences

See Supplementary File
