# Supplementary material for: Weaponizing human EGF-containing fibulin-like extracellular matrix protein 1 (EFEMP1) for 21st century cancer therapeutics
Source: Oncoscience. 2016 May 23;3(7-8):208–19. doi: 10.18632/oncoscience.306 (PMC5043071; doi:10.18632/oncoscience.306)
Supplement: Supplementary file 2 [file oncoscience-03-208-s002.pdf]

Table S1. Multiple alignment of FLAG-tagged EFEMP1 wildtype (E1) and variant (E2 – E18) protein sequences.

```
E1   MDYKDDDDKLKALFLTMLTLALVKSQDTEETITYTQCTDGYEWDPVRQQCKDIDECDIVPDACKGGMKCVNHYGGYLCLPKTAQIIV
E4   MDYKDDDDKLKALFLTMLTLALVKSQDTEETITYTQCTDGYEWDPVRQQCKDIDECDIVPDACKGGMKCVNHYGGYLCLPKTAQIIV
E9   MDYKDDDDKLKALFLTMLTLALVKSQDTEETITYTQCTDGYEWDPVRQQCKDIDECDIVPDACKGGMKCVNHYGGYLCLPKTAQIIV
E10  MDYKDDDDKLKALFLTMLTLALVKSQDTEETITYTQCTDGYEWDPVRQQCKDIDECDIVPDACKGGMKCVNHYGGYLCLPKTAQIIV
E2   MDYKDDDDKLKALFLTMLTLALVKSQDTEETITYTQCTDGYEWDPVRQQCKDIDECDIVPDACKGGMKCVNHYGGYLCLPKTAQIIV
E3   MDYKDDDDKLKALFLTMLTLALVKSQDTEETITYTQCTDGYEWDPVRQQCKDIDECDIVPDACKGGMKCVNHYGGYLCLPKTAQIIV
E5   MDYKDDDDKLKALFLTMLTLALVKSQDTEETITYTQCTDGYEWDPVRQQCKDIDECDIVPDACKGGMKCVNHYGGYLCLPKTAQIIV
E8   MDYKDDDDKLKALFLTMLTLALVKSQDTEETITYTQCTDGYEWDPVRQQCKDIDECDIVPDACKGGMKCVNHYGGYLCLPKTAQIIV
E6   MDYKDDDDKLKALFLTMLTLALVKSQDTEETITYTQCTDGYEWDPVRQQCKDIDECDIVPDACKGGMKCVNHYGGYLCLPKTAQIIV
E7   MDYKDDDDKLKALFLTMLTLALVKSQDTEETITYTQCTDGYEWDPVRQQCKDIDECDIVPDACKGGMKCVNHYGGYLCLPKTAQIIV
E11  MDYKDDDDKLKALFLTMLTLALVKSQDTEETITYTQCTDGYEWDPVRQQCK------------------------------------
E13  MDYKDDDDKLKALFLTMLTLALVKSQDTEETITYTQCTDGYEWDPVRQQCKDIDECDIVPDACKGGMKCVNHYGGYLCLPKTAQIIV
E14  MDYKDDDDKLKALFLTMLTLALVKSQDTEETITYTQCTDGYEWDPVRQQCKDIDECDIVPDACKGGMKCVNHYGGYLCLPKTAQIIV
E15  MDYKDDDDKLKALFLTMLTLALVKSQDTEETITYTQCTDGYEWDPVRQQCKDIDECDIVPDACKGGMKCVNHYGGYLCLPKTAQIIV
E18  MDYKDDDDKLKALFLTMLTLALVKSQDTEETITYTQCTDGYEWDPVRQQCKDIDECDIVPDACKGGMKCVNHYGGYLCLPKTAQIIV

E1   NNEQPQQETQPAEGTSGATTGVVAASSMATSGVLPGGGFVASAAAVAGPEMQTGRNNFVIRRNPADPQRIPSNPSHRIQCAAGYEQS
E4   NNEQPQQETQPAEGTSGATTGVVAASSMATSGVLPGGGFVASAAAVAGPEMQTGRNNFVIRRNPADPQRIPSNPSHRIQCAAGYEQS
E9   NNEQPQQETQPAEGTSGATTGVVAASSMATSGVLPGGGFVASAAAVAGPEMQTGRNNFVIRRNPADPQRIPSNPSHRIQCAAGYEQS
E10  NNEQPQQETQPAEGTSGATTGVVAASSMATSGVLPGGGFVASAAAVAGPEVQTGRNNFVIRRNPADPQRIPSNPSHRIQCAAGYEQS
E2   ---------------------------------------------------------------------------------------
E3   NNEQPQQETQPAEGTSGATTGVVAASSMATSGVLPGGGFVASAAAVAGPEMQTGRNNFVIRRNPADPQRIPSNPSHRIQCAAGYEQS
E5   NNEQPQQETQPAEGTSGATTGVVAASSMATSGVLPGGGFVASAAAVAGPEMQTGRNNFVIRRNPADPQRIPSNPSHRIQCAAGYEQS
E8   NNEQPQQETQPAEGTSGATTGVVAASSMATSGVLPGGGFVASAAAVAGPEMQTGRNNFVIRRNPADPQRIPSNPSHRIQCAAGYEQS
E6   NNEQPQQETQPAEGTSGATTGVVAASSMATSGVLPGGGFVASAAAVAGPEMQTGRNNFVIRRNPADPQRIPSNPSHRIQCAAGYEQS
E7   NNEQPQQETQPAEGTSGATTGVVAASSMATSGVLPGGGFVASAAAVAGPEMQTGRNNFVIRRNPADPQRIPSNPSHRIQCAAGYEQS
E11  ---------------------------------------------------------------------------------------
E13  NNEQPQQETQPAEGTSGATTGVVAASSMATSGVLPGGGFVASAAAVAGPEMQTGRNNFVIRRNPADPQRIPSNPSHRIQCAAGYEQS
E14  NNEQPQQETQPAEGTSGATTGVVAASSMATSGVLPGGGFVASAAAVAGPEMQTGRNNFVIRRNPADPQRIPSNPSHRIQCAAGYEQS
E15  NNEQPQQETQPAEGTSGATTGVVAASSMATSGVLPGGGFVASAAAVAGPEMQTGRNNFVIRRNPADPQRIPSNPSHRIQCAAGYEQS
E18  NNEQPQQETQPAEGTSGATTGVVAASSMATSGVLPGGGFVASAAAVAGPEMQTGRNNFVIRRNPADPQRIPSNPSHRIQCAAGYEQS

E1   EHNVCQDIDECTAGTHNCRADQVCINLRGSFACQCPPGYQKRGEQCVDIDECTIPPYCHQRCVNTPGSFYCQCSPGFQLAANNYTCV
E4   EHNVCQDIDECTAGTHNCRGDQVCINLRGSFACQCPPGYQKRGEQCVDIDECTIPPYCHQRCVNTPGSFYCQCSPGFQLAANNYTCV
E9   EHNVCQDIDECTAGTHNCRGDQVCINLRGSFACQCPPGYQKRGDQCVDIDECTIPPYCHQRCVNTPGSFYCQCSPGFQLAANNYTCV
E10  EHNVCQDIDECTAGTHNCRADQVCINLRGSFACQCPPGYQKRGEQCVDIDECTIPPYCHQRCVNTPGSFYCQCSPGFQLAANNYTCV
E2   ------DIDECTAGTHNCRADQVCINLRGSFACQCPPGYQKRGEQCVDIDECTIPPYCHQRCVNTPGSFYCQCSPGFQLAANNYTCV
E3   EHNVCQDIDECTAGTHNCRADQVCINLRGSFACQCPPGYQKRGEQCVDIDECTIPPYCHQRCVNTPGSFYCQCSPGFQLAANNYTCV
E5   EHNVCQDIDECTAGTHNCRADQVCINLRGSFACQCPPGYQKRGDQCVDIDECTIPPYCHQRCVNTPGSFYCQCSPGFQLAANNYTCV
E8   EHNVCQDIDECTAGTHNCRGDQVCINLRGSFACQCPPGYQKRGEQCVDIDECTIPPYCHQRCVNTPGSFYCQCSPGFQLAANNYTCV
E6   EHNVCK----------------------------------------LDIDECTIPPYCHQRCVNTPGSFYCQCSPGFQLAANNYTCV
E7   EHNVCK----------------------------------------LDIDECTIPPYCHQRCVNTPGSFYCQCSPGFQLAANNYTCV
E11  -----LDIDECTAGTHNCRADQVCINLRGSFACQCPPGYQKRGEQCVDIDECTIPPYCHQRCVNTPGSFYCQCSPGFQLAANNYTCV
E13  EHNVCQDIDECTAGTHNCRADQVCINLRGSFACQCPPGYQKRGEQCV----------------------------------------
E14  EHNVCQDIDECTAGTHNCRGDQVCINLRGSFACQCPPGYQKRGEQCV----------------------------------------
E15  EHNVCQDIDECTAGTHNCRADQVCINLRGSFACQCPPGYQKRGDQCV----------------------------------------
E18  EHNVCQ---------------------------------------------------------------------------------

E1   DINECDASNQCAQQCYNILGSFICQCNQGYELSSDRLNCEDIDECRTSSYLCQYQCVNEPGKFSCMCPQGYQVVRSRTCQDINECET
E4   DINECDASNQCAQQCYNILGSFICQCNQGYELSSDRLNCEDIDECRTSSYLCQYQCVNEPGKFSCMCPQGYQVVRSRTCQDINECET
E9   DINECDASNQCAQQCYNILGSFICQCNQGYELSSDRLNCEDIDECRTSSYLCQYQCVNEPGKFSCMCPQGYQVVRSRTCQDINECET
E10  DINECDASNQCAQQCYNILGSFICQCNQGYELSSDRLNCEDIDECRTSSYLCQYQCVNEPGKFSCMCPQGYQVVRSRTCQDINECET
E2   DINECDASNQCAQQCYNILGSFICQCNQGYELSSDRLNCEDIDECRTSSYLCQYQCVNEPGKFSCMCPQGYQVVRSRTCQDINECET
E3   DINECDASNQCAQQCYNILGSFICQCNQGYELSSDRLNCEDIDECRTSSYLCQYQCVNEPGKFSCMCPQGYQVVRSRTCQDINECET
E5   DINECDASNQCAQQCYNILGSFICQCNQGYELSSDRLNCEDIDECRTSSYLCQYQCVNEPGKFSCMCPQGYQVVRSRTCQDINECET
E8   DINECDASNQCAQQCYNILGSFICQCNQGYELSSDRLNCEDIDECRTSSYLCQYQCVNEPGKFSCMCPQGYQVVRSRTCQDINECET
E6   DINECDASNQCAQQCYNILGSFICQCNQGYELSSDRLNCEDIDECRTSSYLCQYQCVNEPGKFSCMCPQGYQVVRSRTCQDINECET
E7   DINECDASNQCAQQCYNILGSFICQCNQGYELSSDRLNCEDIDECRTSSYLCQYQCVNEPGKFSCMCPQGYQVVRSRTCQDINECET
E11  DINECDASNQCAQQCYNILGSFICQCNQGYELSSDRLNCEDIDECRTSSYLCQYQCVNEPGKFSCMCPQGYQVVRSRTCQDINECET
E13  ---------------------------------------------------------------------------------------
E14  ---------------------------------------------------------------------------------------
E15  ---------------------------------------------------------------------------------------
E18  ---------------------------------------------------------------------------------------
```

```
E1   TNECREDEMCWNYHGGFRCYPRNPCQDPYILTPENRCVCPVSNAMCRELPQSIVYKYMSIRSDRSVPSDIFQIQATTIYANTINTFR
E4   TNECREDEMCWNYHGGFRCYPRNPCQDPYILTPENRCVCPVSNAMCRELPQSIVYKYMSIRSDRSVPSDIFQIQATTIYANTINTFR
E9   TNECREDEMCWNYHGGFRCYPRNPCQDPYILTPENRCVCPVSNAMCRELPQSIVYKYMSIRSDRSVPSDIFQIQATTIYANTINTFR
E10  TNECREDEMCWNYHGGFRCYPRNPCQDPYILTPENRCVCPVSNAMCRELPQSIVYKYMSIRSDRSVPSDIFQIQATTIYANTINTFR
E2   TNECREDEMCWNYHGGFRCYPRNPCQDPYILTPENRCVCPVSNAMCRELPQSIVYKYMSIRSDRSVPSDIFQIQATTIYANTINTFR
E3   TNECREDEMCWNYHGGFRCYPRNPCQDPYILTPENRCV-------------------------------------------------
E5   TNECREDEMCWNYHGGFRCYPRNPCQDPYILTPENRCV-------------------------------------------------
E8   TNECREDEMCWNYHGGFRCYPRNPCQDPYILTPENRCV-------------------------------------------------
E6   TNECREDEMCWNYHGGFRCYPRNPCQDPYILTPENRCV-------------------------------------------------
E7   TNECREDEMCWNYHGGFRCYPRNPCQDPYILTPENRCVCPVSNAMCRELPQSIVYKYMSIRSDRSVPSDIFQIQATTIYANTINTFR
E11  TNECREDEMCWNYHGGFRCYPRNPCQDPYILTPENRCV-------------------------------------------------
E13  ---------------------------------------------------------------------------------------
E14  ---------------------------------------------------------------------------------------
E15  ---------------------------------------------------------------------------------------
E18  ---------------------------------------------------------------------------------------

E1   IKSGNENGEFYLRQTSPVSAMLVLVKSLSGPREHIVDLEMLTVSSIGTFRTSSVLRLTIIVGPFSF    501 aa
E4   IKSGNENGEFYLRQTSPVSAMLVLVKSLSGPREHIVDLEMLTVSSIGTFRTSSVLRLTIIVGPFSF    501 aa
E9   IKSGNENGEFYLRQTSPVSAMLVLVKSLSGPREHIVDLEMLTVSSIGTFRTSSVLRLTIIVGPFSF    501 aa
E10  IKSGNENGEFYLRQTSPVSAMLVLVKSLSGPREHIVDLEMLTVSSIGTFRTSSVLRLTIIVGPFSF    501 aa
E2   IKSGNENGEFYLRQTSPVSAMLVLVKSLSGPREHIVDLEMLTVSSIGTFRTSSVLRLTIIVGPFSF    408 aa
E3   ------------------------------------------------------------------    386 aa
E5   ------------------------------------------------------------------    386 aa
E8   ------------------------------------------------------------------    386 aa
E6   ------------------------------------------------------------------    346 aa
E7   IKSGNENGEFYLRQTSPVSAMLVLVKSLSGPREHIVDLEMLTVSSIGTFRTSSVLRLTIIVGPFSF    461 aa
E11  ------------------------------------------------------------------    258 aa
E13  ------------------------------------------------------------------    221 aa
E14  ------------------------------------------------------------------    221 aa
E15  ------------------------------------------------------------------    221 aa
E18  ------------------------------------------------------------------    180 aa
```

FLAG sequence was underlined in wild-type, mutations and insertions were shown by black and grey highlight, respectively, and deletion by dash. The sequence length was indicated at the end of each sequence.

Table S2. Multiple alignment of FLAG-tagged EFEMP1 wildtype and variant nucleotide sequences.

```
E1     ATGGACTACAAAGACGATGACGATAAATTGAAAGCCCCTTTTCCTAACTATGCTGACTCTGGCGCTGGTCAAGTCACAGGACACCGAAGA
E4     ATGGACTACAAAGACGATGACGATAAATTGAAAGCCCCTTTTCCTAACTATGCTGACTCTGGCGCTGGTCAAGTCACAGGACACCGAAGA
E9     ATGGACTACAAAGACGATGACGATAAATTGAAAGCCCCTTTTCCTAACTATGCTGACTCTGGCGCTGGTCAAGTCACAGGACACCGAAGA
E10    ATGGACTACAAAGACGATGACGATAAATTGAAAGCCCCTTTTCCTAACTATGCTGACTCTGGCGCTGGTCAAGTCACAGGACACCGAAGA
E2     ATGGACTACAAAGACGATGACGATAAATTGAAAGCCCCTTTTCCTAACTATGCTGACTCTGGCGCTGGTCAAGTCACAGGACACCGAAGA
E3     ATGGACTACAAAGACGATGACGATAAATTGAAAGCCCCTTTTCCTAACTATGCTGACTCTGGCGCTGGTCAAGTCACAGGACACCGAAGA
E5     ATGGACTACAAAGACGATGACGATAAATTGAAAGCCCCTTTTCCTAACTATGCTGACTCTGGCGCTGGTCAAGTCACAGGACACCGAAGA
E8     ATGGACTACAAAGACGATGACGATAAATTGAAAGCCCCTTTTCCTAACTATGCTGACTCTGGCGCTGGTCAAGTCACAGGACACCGAAGA
E6     ATGGACTACAAAGACGATGACGATAAATTGAAAGCCCCTTTTCCTAACTATGCTGACTCTGGCGCTGGTCAAGTCACAGGACACCGAAGA
E7     ATGGACTACAAAGACGATGACGATAAATTGAAAGCCCCTTTTCCTAACTATGCTGACTCTGGCGCTGGTCAAGTCACAGGACACCGAAGA
E11    ATGGACTACAAAGACGATGACGATAAATTGAAAGCCCCTTTTCCTAACTATGCTGACTCTGGCGCTGGTCAAGTCACAGGACACCGAAGA
E13    ATGGACTACAAAGACGATGACGATAAATTGAAAGCCCCTTTTCCTAACTATGCTGACTCTGGCGCTGGTCAAGTCACAGGACACCGAAGA
E14    ATGGACTACAAAGACGATGACGATAAATTGAAAGCCCCTTTTCCTAACTATGCTGACTCTGGCGCTGGTCAAGTCACAGGACACCGAAGA
E15    ATGGACTACAAAGACGATGACGATAAATTGAAAGCCCCTTTTCCTAACTATGCTGACTCTGGCGCTGGTCAAGTCACAGGACACCGAAGA
E18    ATGGACTACAAAGACGATGACGATAAATTGAAAGCCCCTTTTCCTAACTATGCTGACTCTGGCGCTGGTCAAGTCACAGGACACCGAAGA

E1     AACCATCACGTACACGCAATGCACTGACGGATATGAGTGGGATCCTGTGAGACAGCAATGCAAAGATATTGATGAATGTGACATTGTCC
E4     AACCATCACGTACACGCAATGCACTGACGGATATGAGTGGGATCCTGTGAGACAGCAATGCAAAGATATTGATGAATGTGACATTGTCC
E9     AACCATCACGTACACGCAATGCACTGACGGATATGAGTGGGATCCTGTGAGACAGCAATGCAAAGATATTGATGAATGTGACATTGTCC
E10    AACCATCACGTACACGCAATGCACTGACGGATATGAGTGGGATCCTGTGAGACAGCAATGCAAAGATATTGATGAATGTGACATTGTCC
E2     AACCATCACGTACACGCAATGCACTGACGGATATGAGTGGGATCCTGTGAGACAGCAATGCAAAGATATTGATGAATGTGACATTGTCC
E3     AACCATCACGTACACGCAATGCACTGACGGATATGAGTGGGATCCTGTGAGACAGCAATGCAAAGATATTGATGAATGTGACATTGTCC
E5     AACCATCACGTACACGCAATGCACTGACGGATATGAGTGGGATCCTGTGAGACAGCAATGCAAAGATATTGATGAATGTGACATTGTCC
E8     AACCATCACGTACACGCAATGCACTGACGGATATGAGTGGGATCCTGTGAGACAGCAATGCAAAGATATTGATGAATGTGACATTGTCC
E6     AACCATCACGTACACGCAATGCACTGACGGATATGAGTGGGATCCTGTGAGACAGCAATGCAAAGATATTGATGAATGTGACATTGTCC
E7     AACCATCACGTACACGCAATGCACTGACGGATATGAGTGGGATCCTGTGAGACAGCAATGCAAAGATATTGATGAATGTGACATTGTCC
E11    AACCATCACGTACACGCAGTGCACTGACGGATATGAGTGGGATCCTGTGAGACAGCAATGCAAG-------------------------
E13    AACCATCACGTACACGCAATGCACTGACGGATATGAGTGGGATCCTGTGAGACAGCAATGCAAAGATATTGATGAATGTGACATTGTCC
E14    AACCATCACGTACACGCAATGCACTGACGGATATGAGTGGGATCCTGTGAGACAGCAATGCAAAGATATTGATGAATGTGACATTGTCC
E15    AACCATCACGTACACGCAATGCACTGACGGATATGAGTGGGATCCTGTGAGACAGCAATGCAAAGATATTGATGAATGTGACATTGTCC
E18    AACCATCACGTACACGCAATGCACTGACGGATATGAGTGGGATCCTGTGAGACAGCAATGCAAAGATATTGATGAATGTGACATTGTCC

E1     CAGACGCTTGTAAAGGTGGAATGAAGTGTGTCAACCACTATGGAGGATACCTCTGCCTTCCGAAAACAGCCCAGATTATTGTCAATAAT
E4     CAGACGCTTGTAAAGGTGGAATGAAGTGTGTCAACCACTATGGAGGATACCTCTGCCTTCCGAAAACAGCCCAGATTATTGTCAATAAT
E9     CAGACGCTTGTAAAGGTGGAATGAAGTGTGTCAACCACTATGGAGGATACCTCTGCCTTCCGAAAACAGCCCAGATTATTGTCAATAAT
E10    CAGACGCTTGTAAAGGTGGAATGAAGTGTGTCAACCACTATGGAGGATACCTCTGCCTTCCGAAAACAGCCCAGATTATTGTCAATAAT
E2     CAGACGCTTGTAAAGGTGGAATGAAGTGTGTCAACCACTATGGAGGATACCTCTGCCTTCCGAAAACAGCCCAGATTATTGTC------
E3     CAGACGCTTGTAAAGGTGGAATGAAGTGTGTCAACCACTATGGAGGATACCTCTGCCTTCCGAAAACAGCCCAGATTATTGTCAATAAT
E5     CAGACGCTTGTAAAGGTGGAATGAAGTGTGTCAACCACTATGGAGGATACCTCTGCCTTCCGAAAACAGCCCAGATTATTGTCAATAAT
E8     CAGACGCTTGTAAAGGTGGAATGAAGTGTGTCAACCACTATGGAGGATACCTCTGCCTTCCGAAAACAGCCCAGATTATTGTCAATAAT
E6     CAGACGCTTGTAAAGGTGGAATGAAGTGTGTCAACCACTATGGAGGATACCTCTGCCTTCCGAAAACAGCCCAGATTATTGTCAATAAT
E7     CAGACGCTTGTAAAGGTGGAATGAAGTGTGTCAACCACTATGGAGGATACCTCTGCCTTCCGAAAACAGCCCAGATTATTGTCAATAAT
E11    -----------------------------------------------------------------------------------------
E13    CAGACGCTTGTAAAGGTGGAATGAAGTGTGTCAACCACTATGGAGGATACCTCTGCCTTCCGAAAACAGCCCAGATTATTGTCAATAAT
E14    CAGACGCTTGTAAAGGTGGAATGAAGTGTGTCAACCACTATGGAGGATACCTCTGCCTTCCGAAAACAGCCCAGATTATTGTCAATAAT
E15    CAGACGCTTGTAAAGGTGGAATGAAGTGTGTCAACCACTATGGAGGATACCTCTGCCTTCCGAAAACAGCCCAGATTATTGTCAATAAT
E18    CAGACGCTTGTAAAGGTGGAATGAAGTGTGTCAACCACTATGGAGGATACCTCTGCCTTCCGAAAACAGCCCAGATTATTGTCAATAAT

E1     GAACAGCCTCAGCAGGAAACACAACCAGCAGAAGGAACCTCAGGGGCAACCACCGGGGTTGTAGCTGCCAGCAGCATGGCAACCAGTGG
E4     GAACAGCCTCAGCAGGAAACACAACCAGCAGAAGGAACCTCAGGGGCAACCACCGGGGTTGTAGCTGCCAGCAGCATGGCAACCAGTGG
E9     GAACAGCCTCAGCAGGAAACACAACCAGCAGAAGGAACCTCAGGGGCAACCACCGGGGTTGTAGCTGCCAGCAGCATGGCAACCAGTGG
E10    GAACAGCCTCAGCAGGAAACACAACCAGCAGAAGGAACCTCAGGGGCAACCACCGGGGTTGTAGCTGCCAGCAGCATGGCAACCAGTGG
E2     -----------------------------------------------------------------------------------------
E3     GAACAGCCTCAGCAGGAAACACAACCAGCAGAAGGAACCTCAGGGGCAACCACCGGGGTTGTAGCTGCCAGCAGCATGGCAACCAGTGG
E5     GAACAGCCTCAGCAGGAAACACAACCAGCAGAAGGAACCTCAGGGGCAACCACCGGGGTTGTAGCTGCCAGCAGCATGGCAACCAGTGG
E8     GAACAGCCTCAGCAGGAAACACAACCAGCAGAAGGAACCTCAGGGGCAACCACCGGGGTTGTAGCTGCCAGCAGCATGGCAACCAGTGG
E6     GAACAGCCTCAGCAGGAAACACAACCAGCAGAAGGAACCTCAGGGGCAACCACCGGGGTTGTAGCTGCCAGCAGCATGGCAACCAGTGG
E7     GAACAGCCTCAGCAGGAAACACAACCAGCAGAAGGAACCTCAGGGGCAACCACCGGGGTTGTAGCTGCCAGCAGCATGGCAACCAGTGG
E11    -----------------------------------------------------------------------------------------
E13    GAACAGCCTCAGCAGGAAACACAACCAGCAGAAGGAACCTCAGGGGCAACCACCGGGGTTGTAGCTGCCAGCAGCATGGCAACCAGTGG
E14    GAACAGCCTCAGCAGGAAACACAACCAGCAGAAGGAACCTCAGGGGCAACCACCGGGGTTGTAGCTGCCAGCAGCATGGCAACCAGTGG
E15    GAACAGCCTCAGCAGGAAACACAACCAGCAGAAGGAACCTCAGGGGCAACCACCGGGGTTGTAGCTGCCAGCAGCATGGCAACCAGTGG
E18    GAACAGCCTCAGCAGGAAACACAACCAGCAGAAGGAACCTCAGGGGCAACCACCGGGGTTGTAGCTGCCAGCAGCATGGCAACCAGTGG
```

```
E1    AGTGTTGCCCGGGGGTGGTTTTGTGGCCAGTGCTGCTGCAGTCGCAGGCCCTGAAATGCAGACTGGCCGAAATAACTTTGTCATCCGGC
E4    AGTGTTGCCCGGGGGTGGTTTTGTGGCCAGTGCTGCTGCAGTCGCAGGCCCTGAAATGCAGACTGGCCGAAATAACTTTGTCATCCGGC
E9    AGTGTTGCCCGGGGGTGGTTTTGTGGCCAGTGCTGCTGCAGTCGCAGGCCCTGAAATGCAGACTGGCCGAAATAACTTTGTCATCCGGC
E10   AGTGTTGCCCGGGGGTGGTTTTGTGGCCAGTGCTGCTGCAGTCGCAGGCCCTGAAGTGCAGACTGGCCGAAATAACTTTGTCATCCGGC
E2    -----------------------------------------------------------------------------------------
E3    AGTGTTGCCCGGGGGTGGTTTTGTGGCCAGTGCTGCTGCAGTCGCAGGCCCTGAAATGCAGACTGGCCGAAATAACTTTGTCATCCGGC
E5    AGTGTTGCCCGGGGGTGGTTTTGTGGCCAGTGCTGCTGCAGTCGCAGGCCCTGAAATGCAGACTGGCCGAAATAACTTTGTCATCCGGC
E8    AGTGTTGCCCGGGGGTGGTTTTGTGGCCAGTGCTGCTGCAGTCGCAGGCCCTGAAATGCAGACTGGCCGAAATAACTTTGTCATCCGGC
E6    AGTGTTGCCCGGGGGTGGTTTTGTGGCCAGTGCTGCTGCAGTCGCAGGCCCTGAAATGCAGACTGGCCGAAATAACTTTGTCATCCGGC
E7    AGTGTTGCCCGGGGGTGGTTTTGTGGCCAGTGCTGCTGCAGTCGCAGGCCCTGAAATGCAGACTGGCCGAAATAACTTTGTCATCCGGC
E11   -----------------------------------------------------------------------------------------
E13   AGTGTTGCCCGGGGGTGGTTTTGTGGCCAGTGCTGCTGCAGTCGCAGGCCCTGAAATGCAGACTGGCCGAAATAACTTTGTCATCCGGC
E14   AGTGTTGCCCGGGGGTGGTTTTGTGGCCAGTGCTGCTGCAGTCGCAGGCCCTGAAATGCAGACTGGCCGAAATAACTTTGTCATCCGGC
E15   AGTGTTGCCCGGGGGTGGTTTTGTGGCCAGTGCTGCTGCAGTCGCAGGCCCTGAAATGCAGACTGGCCGAAATAACTTTGTCATCCGGC
E18   AGTGTTGCCCGGGGGTGGTTTTGTGGCCAGTGCTGCTGCAGTCGCAGGCCCTGAAATGCAGACTGGCCGAAATAACTTTGTCATCCGGC

E1    GGAACCCAGCTGACCCTCAGCGCATTCCCTCCAACCCTTCCCACCGTATCCAGTGTGCAGCAGGCTACGAGCAAAGTGAACACAACGTG
E4    GGAACCCAGCTGACCCTCAGCGCATTCCCTCCAACCCTTCCCACCGTATCCAGTGTGCAGCAGGCTACGAGCAAAGTGAACACAACGTG
E9    GGAACCCAGCTGACCCTCAGCGCATTCCCTCCAACCCTTCCCACCGTATCCAGTGTGCAGCAGGCTACGAGCAAAGTGAACACAACGTG
E10   GGAACCCAGCTGACCCTCAGCGCATTCCCTCCAACCCTTCCCACCGTATCCAGTGTGCAGCAGGCTACGAGCAAAGTGAACACAACGTG
E2    -----------------------------------------------------------------------------------------
E3    GGAACCCAGCTGACCCTCAGCGCATTCCCTCCAACCCTTCCCACCGTATCCAGTGTGCAGCAGGCTACGAGCAAAGTGAACACAACGTG
E5    GGAACCCAGCTGACCCTCAGCGCATTCCCTCCAACCCTTCCCACCGTATCCAGTGTGCAGCAGGCTACGAGCAAAGTGAACACAACGTG
E8    GGAACCCAGCTGACCCTCAGCGCATTCCCTCCAACCCTTCCCACCGTATCCAGTGTGCAGCAGGCTACGAGCAAAGTGAACACAACGTG
E6    GGAACCCAGCTGACCCTCAGCGCATTCCCTCCAACCCTTCCCACCGTATCCAGTGTGCAGCAGGCTACGAGCAAAGTGAACACAACGTG
E7    GGAACCCAGCTGACCCTCAGCGCATTCCCTCCAACCCTTCCCACCGTATCCAGTGTGCAGCAGGCTACGAGCAAAGTGAACACAACGTG
E11   -----------------------------------------------------------------------------------------
E13   GGAACCCAGCTGACCCTCAGCGCATTCCCTCCAACCCTTCCCACCGTATCCAGTGTGCAGCAGGCTACGAGCAAAGTGAACACAACGTG
E14   GGAACCCAGCTGACCCTCAGCGCATTCCCTCCAACCCTTCCCACCGTATCCAGTGTGCAGCAGGCTACGAGCAAAGTGAACACAACGTG
E15   GGAACCCAGCTGACCCTCAGCGCATTCCCTCCAACCCTTCCCACCGTATCCAGTGTGCAGCAGGCTACGAGCAAAGTGAACACAACGTG
E18   GGAACCCAGCTGACCCTCAGCGCATTCCCTCCAACCCTTCCCACCGTATCCAGTGTGCAGCAGGCTACGAGCAAAGTGAACACAACGTG

E1    TGCCAA---GACATAGACGAGTGCACTGCAGGGACGCACAACTGTAGAGCAGACCAAGTGTGCATCAATTTACGGGGATCCTTTGCATG
E4    TGCCAA---GACATAGACGAGTGCACTGCAGGGACGCACAACTGTAGAGGAGACCAAGTGTGCATCAATTTACGGGGATCCTTTGCATG
E9    TGCCAA---GACATAGACGAGTGCACTGCAGGGACGCACAACTGTAGAGGAGACCAAGTGTGCATCAATTTACGGGGATCCTTTGCATG
E10   TGCCAA---GACATAGACGAGTGCACTGCAGGGACGCACAACTGTAGAGCAGACCAAGTGTGCATCAATTTACGGGGATCCTTTGCATG
E2    ---------GACATAGACGAGTGCACTGCAGGGACGCACAACTGTAGAGCAGACCAAGTGTGCATCAATTTACGGGGATCCTTTGCATG
E3    TGCCAA---GACATAGACGAGTGCACTGCAGGGACGCACAACTGTAGAGCAGACCAAGTGTGCATCAATTTACGGGGATCCTTTGCATG
E5    TGCCAA---GACATAGACGAGTGCACTGCAGGGACGCACAACTGTAGAGCAGACCAAGTGTGCATCAATTTACGGGGATCCTTTGCATG
E8    TGCCAA---GACATAGACGAGTGCACTGCAGGGACGCACAACTGTAGAGGAGACCAAGTGTGCATCAATTTACGGGGATCCTTTGCATG
E6    TGCAAGCTT--------------------------------------------------------------------------------
E7    TGCAAGCTT--------------------------------------------------------------------------------
E11   ------CTTGACATAGACGAGTGCACTGCAGGGACGCACAACTGTAGAGCAGACCAAGTGTGCATCAATTTACGGGGATCCTTTGCATG
E13   TGCCAA---GACATAGACGAGTGCACTGCAGGGACGCACAACTGTAGAGCAGACCAAGTGTGCATCAATTTACGGGGATCCTTTGCATG
E14   TGCCAA---GACATAGACGAGTGCACTGCAGGGACGCACAACTGTAGAGGAGACCAAGTGTGCATCAATTTACGGGGATCCTTTGCATG
E15   TGCCAA---GACATAGACGAGTGCACTGCAGGGACGCACAACTGTAGAGCAGACCAAGTGTGCATCAATTTACGGGGATCCTTTGCATG
E18   TGCCAATAA--------------------------------------------------------------------------------

E1    TCAGTGCCCTCCTGGATATCAGAAGCGAGGGGAGCAGTGCGTAGACATAGATGAATGTACCATCCCTCCATATTGCCACCAAAGATGCG
E4    TCAGTGCCCTCCTGGATATCAGAAGCGAGGGGAGCAGTGCGTAGACATAGATGAATGTACCATCCCTCCATATTGCCACCAAAGATGCG
E9    TCAGTGCCCTCCTGGATATCAGAAGCGAGGGGACCAGTGCGTAGACATAGATGAATGTACCATCCCTCCATATTGCCACCAAAGATGCG
E10   TCAGTGCCCTCCTGGATATCAGAAGCGAGGGGAGCAGTGCGTAGACATAGATGAATGTACCATCCCTCCATATTGCCACCAAAGATGCG
E2    TCAGTGCCCTCCTGGATATCAGAAGCGAGGGGAGCAGTGCGTAGACATAGATGAATGTACCATCCCTCCATATTGCCACCAAAGATGCG
E3    TCAGTGCCCTCCTGGATATCAGAAGCGAGGGGAGCAGTGCGTAGACATAGATGAATGTACCATCCCTCCATATTGCCACCAAAGATGCG
E5    TCAGTGCCCTCCTGGATATCAGAAGCGAGGGGACCAGTGCGTAGACATAGATGAATGTACCATCCCTCCATATTGCCACCAAAGATGCG
E8    TCAGTGCCCTCCTGGATATCAGAAGCGAGGGGAGCAGTGCGTAGACATAGATGAATGTACCATCCCTCCATATTGCCACCAAAGATGCG
E6    -------------------------------------------GACATAGATGAATGTACCATCCCTCCATATTGCCACCAAAGATGCG
E7    -------------------------------------------GACATAGATGAATGTACCATCCCTCCATATTGCCACCAAAGATGCG
E11   TCAGTGCCCTCCTGGATATCAGAAGCGAGGGGAGCAGTGCGTAGACATAGATGAATGTACCATCCCTCCATATTGCCACCAAAGATGCG
E13   TCAGTGCCCTCCTGGATATCAGAAGCGAGGGGAGCAGTGCGTATAG-------------------------------------------
E14   TCAGTGCCCTCCTGGATATCAGAAGCGAGGGGAGCAGTGCGTATAG-------------------------------------------
E15   TCAGTGCCCTCCTGGATATCAGAAGCGAGGGGACCAGTGCGTATAG-------------------------------------------
E18   -----------------------------------------------------------------------------------------
```

```
E1     TGAATACACCAGGCTCATTTTATTGCCAGTGCAGTCCTGGGTTTCAATTGGCAGCAAACAACTATACCTGCGTAGATATAAATGAATGT
E4     TGAATACACCAGGCTCATTTTATTGCCAGTGCAGTCCTGGGTTTCAATTGGCAGCAAACAACTATACCTGCGTAGATATAAATGAATGT
E9     TGAATACACCAGGCTCATTTTATTGCCAGTGCAGTCCTGGGTTTCAATTGGCAGCAAACAACTATACCTGCGTAGATATAAATGAATGT
E10    TGAATACACCAGGCTCATTTTATTGCCAGTGCAGTCCTGGGTTTCAATTGGCAGCAAACAACTATACCTGCGTAGATATAAATGAATGT
E2     TGAATACACCAGGCTCATTTTATTGCCAGTGCAGTCCTGGGTTTCAATTGGCAGCAAACAACTATACCTGCGTAGATATAAATGAATGT
E3     TGAATACACCAGGCTCATTTTATTGCCAGTGCAGTCCTGGGTTTCAATTGGCAGCAAACAACTATACCTGCGTAGATATAAATGAATGT
E5     TGAATACACCAGGCTCATTTTATTGCCAGTGCAGTCCTGGGTTTCAATTGGCAGCAAACAACTATACCTGCGTAGATATAAATGAATGT
E8     TGAATACACCAGGCTCATTTTATTGCCAGTGCAGTCCTGGGTTTCAATTGGCAGCAAACAACTATACCTGCGTAGATATAAATGAATGT
E6     TGAATACACCAGGCTCATTTTATTGCCAGTGCAGTCCTGGGTTTCAATTGGCAGCAAACAACTATACCTGCGTAGATATAAATGAATGT
E7     TGAATACACCAGGCTCATTTTATTGCCAGTGCAGTCCTGGGTTTCAATTGGCAGCAAACAACTATACCTGCGTAGATATAAATGAATGT
E11    TGAATACACCAGGCTCATTTTATTGCCAGTGCAGTCCTGGGTTTCAATTGGCAGCAAACAACTATACCTGCGTAGATATAAATGAATGT
E13    -----------------------------------------------------------------------------------------
E14    -----------------------------------------------------------------------------------------
E15    -----------------------------------------------------------------------------------------
E18    -----------------------------------------------------------------------------------------

E1     GATGCCAGCAATCAATGTGCTCAGCAGTGCTACAACATTCTTGGTTCATTCATCTGTCAGTGCAATCAAGGATATGAGCTAAGCAGTGA
E4     GATGCCAGCAATCAATGTGCTCAGCAGTGCTACAACATTCTTGGTTCATTCATCTGTCAGTGCAATCAAGGATATGAGCTAAGCAGTGA
E9     GATGCCAGCAATCAATGTGCTCAGCAGTGCTACAACATTCTTGGTTCATTCATCTGTCAGTGCAATCAAGGATATGAGCTAAGCAGTGA
E10    GATGCCAGCAATCAATGTGCTCAGCAGTGCTACAACATTCTTGGTTCATTCATCTGTCAGTGCAATCAAGGATATGAGCTAAGCAGTGA
E2     GATGCCAGCAATCAATGTGCTCAGCAGTGCTACAACATTCTTGGTTCATTCATCTGTCAGTGCAATCAAGGATATGAGCTAAGCAGTGA
E3     GATGCCAGCAATCAATGTGCTCAGCAGTGCTACAACATTCTTGGTTCATTCATCTGTCAGTGCAATCAAGGATATGAGCTAAGCAGTGA
E5     GATGCCAGCAATCAATGTGCTCAGCAGTGCTACAACATTCTTGGTTCATTCATCTGTCAGTGCAATCAAGGATATGAGCTAAGCAGTGA
E8     GATGCCAGCAATCAATGTGCTCAGCAGTGCTACAACATTCTTGGTTCATTCATCTGTCAGTGCAATCAAGGATATGAGCTAAGCAGTGA
E6     GATGCCAGCAATCAATGTGCTCAGCAGTGCTACAACATTCTTGGTTCATTCATCTGTCAGTGCAATCAAGGATATGAGCTAAGCAGTGA
E7     GATGCCAGCAATCAATGTGCTCAGCAGTGCTACAACATTCTTGGTTCATTCATCTGTCAGTGCAATCAAGGATATGAGCTAAGCAGTGA
E11    GATGCCAGCAATCAATGTGCTCAGCAGTGCTACAACATTCTTGGTTCATTCATCTGTCAGTGCAATCAAGGATATGAGCTAAGCAGTGA
E13    -----------------------------------------------------------------------------------------
E14    -----------------------------------------------------------------------------------------
E15    -----------------------------------------------------------------------------------------
E18    -----------------------------------------------------------------------------------------

E1     CAGGCTCAACTGTGAAGACATTGATGAATGCAGAACCTCAAGCTACCTGTGTCAATATCAATGTGTCAATGAACCTGGGAAATTCTCAT
E4     CAGGCTCAACTGTGAAGACATTGATGAATGCAGAACCTCAAGCTACCTGTGTCAATATCAATGTGTCAATGAACCTGGGAAATTCTCAT
E9     CAGGCTCAACTGTGAAGACATTGATGAATGCAGAACCTCAAGCTACCTGTGTCAATATCAATGTGTCAATGAACCTGGGAAATTCTCAT
E10    CAGGCTCAACTGTGAAGACATTGATGAATGCAGAACCTCAAGCTACCTGTGTCAATATCAATGTGTCAATGAACCTGGGAAATTCTCAT
E2     CAGGCTCAACTGTGAAGACATTGATGAATGCAGAACCTCAAGCTACCTGTGTCAATATCAATGTGTCAATGAACCTGGGAAATTCTCAT
E3     CAGGCTCAACTGTGAAGACATTGATGAATGCAGAACCTCAAGCTACCTGTGTCAATATCAATGTGTCAATGAACCTGGGAAATTCTCAT
E5     CAGGCTCAACTGTGAAGACATTGATGAATGCAGAACCTCAAGCTACCTGTGTCAATATCAATGTGTCAATGAACCTGGGAAATTCTCAT
E8     CAGGCTCAACTGTGAAGACATTGATGAATGCAGAACCTCAAGCTACCTGTGTCAATATCAATGTGTCAATGAACCTGGGAAATTCTCAT
E6     CAGGCTCAACTGTGAAGACATTGATGAATGCAGAACCTCAAGCTACCTGTGTCAATATCAATGTGTCAATGAACCTGGGAAATTCTCAT
E7     CAGGCTCAACTGTGAAGACATTGATGAATGCAGAACCTCAAGCTACCTGTGTCAATATCAATGTGTCAATGAACCTGGGAAATTCTCAT
E11    CAGGCTCAACTGTGAAGACATTGATGAATGCAGAACCTCAAGCTACCTGTGTCAATATCAATGTGTCAATGAACCTGGGAAATTCTCAT
E13    -----------------------------------------------------------------------------------------
E14    -----------------------------------------------------------------------------------------
E15    -----------------------------------------------------------------------------------------
E18    -----------------------------------------------------------------------------------------

E1     GTATGTGCCCCCAGGGATACCAAGTGGTGAGAAGTAGAACATGTCAAGATATAAATGAGTGTGAGACCACAAATGAATGCCGGGAGGAT
E4     GTATGTGCCCCCAGGGATACCAAGTGGTGAGAAGTAGAACATGTCAAGATATAAATGAGTGTGAGACCACAAATGAATGCCGGGAGGAT
E9     GTATGTGCCCCCAGGGATACCAAGTGGTGAGAAGTAGAACATGTCAAGATATAAATGAGTGTGAGACCACAAATGAATGCCGGGAGGAT
E10    GTATGTGCCCCCAGGGATACCAAGTGGTGAGAAGTAGAACATGTCAAGATATAAATGAGTGTGAGACCACAAATGAATGCCGGGAGGAT
E2     GTATGTGCCCCCAGGGATACCAAGTGGTGAGAAGTAGAACATGTCAAGATATAAATGAGTGTGAGACCACAAATGAATGCCGGGAGGAT
E3     GTATGTGCCCCCAGGGATACCAAGTGGTGAGAAGTAGAACATGTCAAGATATAAATGAGTGTGAGACCACAAATGAATGCCGGGAGGAT
E5     GTATGTGCCCCCAGGGATACCAAGTGGTGAGAAGTAGAACATGTCAAGATATAAATGAGTGTGAGACCACAAATGAATGCCGGGAGGAT
E8     GTATGTGCCCCCAGGGATACCAAGTGGTGAGAAGTAGAACATGTCAAGATATAAATGAGTGTGAGACCACAAATGAATGCCGGGAGGAT
E6     GTATGTGCCCCCAGGGATACCAAGTGGTGAGAAGTAGAACATGTCAAGATATAAATGAGTGTGAGACCACAAATGAATGCCGGGAGGAT
E7     GTATGTGCCCCCAGGGATACCAAGTGGTGAGAAGTAGAACATGTCAAGATATAAATGAGTGTGAGACCACAAATGAATGCCGGGAGGAT
E11    GTATGTGCCCCCAGGGATACCAAGTGGTGAGAAGTAGAACATGTCAAGATATAAATGAGTGTGAGACCACAAATGAATGCCGGGAGGAT
E13    -----------------------------------------------------------------------------------------
E14    -----------------------------------------------------------------------------------------
E15    -----------------------------------------------------------------------------------------
E18    -----------------------------------------------------------------------------------------
```

```
E1     GAAATGTGTTGGAATTATCATGGCGGCTTCCGTTGTTATCCACGAAATCCTTGTCAAGATCCCTACATTCTAACACCAGAGAACCGATG
E4     GAAATGTGTTGGAATTATCATGGCGGCTTCCGTTGTTATCCACGAAATCCTTGTCAAGATCCCTACATTCTAACACCAGAGAACCGATG
E9     GAAATGTGTTGGAATTATCATGGCGGCTTCCGTTGTTATCCACGAAATCCTTGTCAAGATCCCTACATTCTAACACCAGAGAACCGATG
E10    GAAATGTGTTGGAATTATCATGGCGGCTTCCGTTGTTATCCACGAAATCCTTGTCAAGATCCCTACATTCTAACACCAGAGAACCGATG
E2     GAAATGTGTTGGAATTATCATGGCGGCTTCCGTTGTTATCCACGAAATCCTTGTCAAGATCCCTACATTCTAACACCAGAGAACCGATG
E3     GAAATGTGTTGGAATTATCATGGCGGCTTCCGTTGTTATCCACGAAATCCTTGTCAAGATCCCTACATTCTAACACCAGAGAACCGATG
E5     GAAATGTGTTGGAATTATCATGGCGGCTTCCGTTGTTATCCACGAAATCCTTGTCAAGATCCCTACATTCTAACACCAGAGAACCGATG
E8     GAAATGTGTTGGAATTATCATGGCGGCTTCCGTTGTTATCCACGAAATCCTTGTCAAGATCCCTACATTCTAACACCAGAGAACCGATG
E6     GAAATGTGTTGGAATTATCATGGCGGCTTCCGTTGTTATCCACGAAATCCTTGTCAAGATCCCTACATTCTAACACCAGAGAACCGATG
E7     GAAATGTGTTGGAATTATCATGGCGGCTTCCGTTGTTATCCACGAAATCCTTGTCAAGATCCCTACATTCTAACACCAGAGAACCGATG
E11    GAAATGTGTTGGAATTATCATGGCGGCTTCCGTTGTTATCCACGAAATCCTTGTCAAGATCCCTACATTCTAACACCAGAGAACCGATG
E13    -----------------------------------------------------------------------------------------
E14    -----------------------------------------------------------------------------------------
E15    -----------------------------------------------------------------------------------------
E18    -----------------------------------------------------------------------------------------

E1     TGTTTGCCCAGTCTCAAATGCCATGTGCCGAGAACTGCCCCAGTCAATAGTCTACAAATACATGAGCATCCGATCTGATAGGTCTGTGC
E4     TGTTTGCCCAGTCTCAAATGCCATGTGCCGAGAACTGCCCCAGTCAATAGTCTACAAATACATGAGCATCCGATCTGATAGGTCTGTGC
E9     TGTTTGCCCAGTCTCAAATGCCATGTGCCGAGAACTGCCCCAGTCAATAGTCTACAAATACATGAGCATCCGATCTGATAGGTCTGTGC
E10    TGTTTGCCCAGTCTCAAATGCCATGTGCCGAGAACTGCCCCAGTCAATAGTCTACAAATACATGAGCATCCGATCTGATAGGTCTGTGC
E2     TGTTTGCCCAGTCTCAAATGCCATGTGCCGAGAACTGCCCCAGTCAATAGTCTACAAATACATGAGCATCCGATCTGATAGGTCTGTGC
E3     TGTTTAA----------------------------------------------------------------------------------
E5     TGTTTAA----------------------------------------------------------------------------------
E8     TGTTTAA----------------------------------------------------------------------------------
E6     TGTTTAA----------------------------------------------------------------------------------
E7     TGTTTGCCCAGTCTCAAATGCCATGTGCCGAGAACTGCCCCAGTCAATAGTCTACAAATACATGAGCATCCGATCTGATAGGTCTGTGC
E11    TGTTTAA----------------------------------------------------------------------------------
E13    -----------------------------------------------------------------------------------------
E14    -----------------------------------------------------------------------------------------
E15    -----------------------------------------------------------------------------------------
E18    -----------------------------------------------------------------------------------------

E1     CATCAGACATCTTCCAGATACAGGCCACAACTATTTATGCCAACACCATCAATACTTTTCGGATTAAATCTGGAAATGAAAATGGAGAG
E4     CATCAGACATCTTCCAGATACAGGCCACAACTATTTATGCCAACACCATCAATACTTTTCGGATTAAATCTGGAAATGAAAATGGAGAG
E9     CATCAGACATCTTCCAGATACAGGCCACAACTATTTATGCCAACACCATCAATACTTTTCGGATTAAATCTGGAAATGAAAATGGAGAG
E10    CATCAGACATCTTCCAGATACAGGCCACAACTATTTATGCCAACACCATCAATACTTTTCGGATTAAATCTGGAAATGAAAATGGAGAG
E2     CATCAGACATCTTCCAGATACAGGCCACAACTATTTATGCCAACACCATCAATACTTTTCGGATTAAATCTGGAAATGAAAATGGAGAG
E3     -----------------------------------------------------------------------------------------
E5     -----------------------------------------------------------------------------------------
E8     -----------------------------------------------------------------------------------------
E6     -----------------------------------------------------------------------------------------
E7     CATCAGACATCTTCCAGATACAGGCCACAACTATTTATGCCAACACCATCAATACTTTTCGGATTAAATCTGGAAATGAAAATGGAGAG
E11    -----------------------------------------------------------------------------------------
E13    -----------------------------------------------------------------------------------------
E14    -----------------------------------------------------------------------------------------
E15    -----------------------------------------------------------------------------------------
E18    -----------------------------------------------------------------------------------------

E1     TTCTACCTACGACAAACAAGTCCTGTAAGTGCAATGCTTGTGCTCGTGAAGTCATTATCAGGACCAAGAGAACATATCGTGGACCTGGA
E4     TTCTACCTACGACAAACAAGTCCTGTAAGTGCAATGCTTGTGCTCGTGAAGTCATTATCAGGACCAAGAGAACATATCGTGGACCTGGA
E9     TTCTACCTACGACAAACAAGTCCTGTAAGTGCAATGCTTGTGCTCGTGAAGTCATTATCAGGACCAAGAGAACATATCGTGGACCTGGA
E10    TTCTACCTACGACAAACAAGTCCTGTAAGTGCAATGCTTGTGCTCGTGAAGTCATTATCAGGACCAAGAGAACATATCGTGGACCTGGA
E2     TTCTACCTACGACAAACAAGTCCTGTAAGTGCAATGCTTGTGCTCGTGAAGTCATTATCAGGACCAAGAGAACATATCGTGGACCTGGA
E3     -----------------------------------------------------------------------------------------
E5     -----------------------------------------------------------------------------------------
E8     -----------------------------------------------------------------------------------------
E6     -----------------------------------------------------------------------------------------
E7     TTCTACCTACGACAAACAAGTCCTGTAAGTGCAATGCTTGTGCTCGTGAAGTCATTATCAGGACCAAGAGAACATATCGTGGACCTGGA
E11    -----------------------------------------------------------------------------------------
E13    -----------------------------------------------------------------------------------------
E14    -----------------------------------------------------------------------------------------
E15    -----------------------------------------------------------------------------------------
E18    -----------------------------------------------------------------------------------------
```

```
E1    GATGCTGACAGTCAGCAGTATAGGGACCTTCCGCACAAGCTCTGTGTTAAGATTGACAATAATAGTGGGGCCATTTTCATTTTAG 1506 bp
E4    GATGCTGACAGTCAGCAGTATAGGGACCTTCCGCACAAGCTCTGTGTTAAGATTGACAATAATAGTGGGGCCATTTTCATTTTAG 1506 bp
E9    GATGCTGACAGTCAGCAGTATAGGGACCTTCCGCACAAGCTCTGTGTTAAGATTGACAATAATAGTGGGGCCATTTTCATTTTAG 1506 bp
E10   GATGCTGACAGTCAGCAGTATAGGGACCTTCCGCACAAGCTCTGTGTTAAGATTGACAATAATAGTGGGGCCATTTTCATTTTAG 1506 bp
E2    GATGCTGACAGTCAGCAGTATAGGGACCTTCCGCACAAGCTCTGTGTTAAGATTGACAATAATAGTGGGGCCATTTTCATTTTAG 1227 bp
E3    ------------------------------------------------------------------------------------- 1161 bp
E5    ------------------------------------------------------------------------------------- 1161 bp
E8    ------------------------------------------------------------------------------------- 1161 bp
E6    ------------------------------------------------------------------------------------- 1041 bp
E7    GATGCTGACAGTCAGCAGTATAGGGACCTTCCGCACAAGCTCTGTGTTAAGATTGACAATAATAGTGGGGCCATTTTCATTTTAG 1386 bp
E11   ------------------------------------------------------------------------------------- 777 bp
E13   ------------------------------------------------------------------------------------- 666 bp
E14   ------------------------------------------------------------------------------------- 666 bp
E15   ------------------------------------------------------------------------------------- 666 bp
E18   ------------------------------------------------------------------------------------- 543 bp
```

FLAG sequence was underlined in wild-type, mutations and insertions were shown by black and grey highlight, respectively, deletion by dash, and stop codon by boldface. The sequence length was indicated at the end of each sequence.
